# Genetic Artificial Hummingbird Algorithm-Support Vector Machine for Timely Power Theft Detection

**DOI:** 10.1155/2024/5568922

**Published:** 2024-09-02

**Authors:** Emmanuel Gbafore, Davies Rene Segera, Cosmas Raymond Mutugi Kiruki

**Affiliations:** Department of Electrical and Information Engineering University of Nairobi, Nairobi 301971, Kenya

## Abstract

Utilities face serious obstacles from power theft, which calls for creative ways to maintain income and improve operational effectiveness. This study presents a novel hybrid genetic artificial hummingbird algorithm-support vector machine classifier to detect power theft. The proposed algorithm combines the artificial hummingbird algorithm exploration phase with the genetic algorithm's mutation and crossover operators, to optimize the support vector machine's hyperparameters and categorize users as fraudulent or nonfraudulent. It utilizes 7,270 rows of labeled historical electricity consumption data from the Liberia Electricity Corporation over 15 independent runs. The methodology entailed data preprocessing, data split into training, validation, and testing sets in an 80-10-10 ratio, z-score normalization, optimization, training, validation, testing, and computation of six evaluation metrics. Its performance is compared with 13 metaheuristic classifiers and the conventional support vector machine. Findings indicate that the genetic artificial hummingbird algorithm-support vector machine outperforms the 13 rivals and the standard support vector machine in the six assessment measures with an accuracy score of 0.9986, precision of 0.9971, *f*_score of 0.9986, recall of 1, Matthews correlation coefficient of 0.9972, and *g*_mean of 0.9987. Furthermore, 90% of the time, Wilcoxon rank-sum tests revealed statistically significant differences between the algorithm and its rivals, demonstrating its superiority. The average run time is 4,656 seconds, the 3^rd^ highest among its competitors. Despite the time complexity trade-off, its excellent performance on the unimodal and multimodal benchmark test functions, placing joint best in 7 out of 7 and 5 out of 6, respectively, provides important insights into the model's capacity to balance exploitation and exploration, improve local search, and avoid becoming stuck in the local optimum. These findings address important metaheuristic optimization gaps highlighting the model's potential for power theft detection.

## 1. Introduction

In recent years, addressing electricity fraud detection has garnered significant attention as researchers strive to devise robust models for identifying power theft within power grids. The ramifications of power theft encompass substantial nontechnical losses, including revenue depletion, compromised service quality, and even potential fire hazards [1]. This issue is pronounced in countries like Liberia, where electricity access rates remain low, with approximately 12% lacking access and an alarming 60% of generated electricity siphoned off through illegal connections [2]. The existing approach to power theft detection by the Liberia Electricity Corporation (LEC) hinges on manual inspections, a process beset by time constraints and logistical challenges. Despite enacting power theft legislation in 2019, the persistence of power theft underscores the need for a more effective solution that proactively identifies culprits.

Worldwide, diverse strategies encompassing state-based, game theory-based, and artificial intelligence-based methodologies are explored to tackle power theft. While state-based solutions employ sensors to monitor grid states, their costliness hinders widespread adoption. Game theory-based approaches introduce complexity and can result in a surplus of false positives. The emergence of artificial intelligence methods, mainly supervised learning techniques, has garnered attention due to their superior results and cost-effectiveness [3, 4].

However, current AI-driven approaches frequently encounter challenges such as overfitting, poor handling of class imbalance, and suboptimal hyperparameter tuning, which limits their performance in practical scenarios. Current literature underscores the need for more effective optimization of machine learning models for fraud detection in electricity grids, particularly in overcoming the limitations of existing AI-based approaches, requiring a method that prevents overfitting, addresses class imbalance, and optimizes hyperparameters to significantly enhance model performance. Conventional optimization techniques such as grid search are frequently computationally intensive and may not deliver optimal results. Hence, there is a compelling need for a novel approach that leverages the strengths of advanced metaheuristic algorithms for enhanced optimization.

In this context, this study introduces an innovative approach by combining the genetic algorithm's operators and the artificial hummingbird algorithm to optimize the support vector machine for power theft detection. The genetic algorithm (GA) is known for its robust exploration capabilities and ability to prevent premature convergence through its use of crossover and mutation operators, while the artificial hummingbird algorithm (AHA) excels in exploitation and local search tasks by mimicking the foraging mechanisms of hummingbirds. By merging these algorithms, a balance between exploration and exploitation is struck, ultimately enhancing the optimization process. This approach aligns with Wolpert's no free lunch theorem, which states that no single algorithm is universally optimal for all optimization problems [5].

Support vector machine (SVM) is the choice of classifier due to its effectiveness in handling high-dimensional spaces and its robustness against overfitting. SVM's ability to draw a clear boundary between fraudulent and nonfraudulent users makes it well suited for binary classification problems such as fraud detection. While algorithms like XGBoost or AdaBoost are powerful, SVM's capability to handle nonlinear relationships through the kernel trick makes it a fitting choice for this problem. Additionally, SVM's proven performance in similar applications provides a solid foundation for this hybrid model.

The primary objective of this study is to optimize SVM hyperparameters using the GAHA approach, thereby enhancing the classification accuracy for fraud detection. Specifically, it aims to evaluate the GAHA-SVM model's performance using a comprehensive suite of evaluation metrics, including accuracy, precision, recall, *f*_score, MCC, and G_mean, and compare it against a spectrum of 13 bio-inspired classifiers and the traditional SVM grid search. Subsequent analysis answers the research questions: (1) How does the hybrid genetic artificial hummingbird algorithm compare to the conventional SVM grid search method in optimizing SVM's C and gamma hyperparameters? (2) To what extent do genetic algorithm mutation operators enhance the exploration capabilities of the artificial hummingbird algorithm in achieving better optimization results? (3) What are the performance metrics such as accuracy, precision, *f*_score, recall, MCC, and gscore for the proposed algorithm, and how do they reflect its effectiveness? (4) In what ways does the hybrid genetic artificial hummingbird algorithm outperform or underperform in comparison to the 13 other classifier algorithms (particle swarm optimization [6], grey wolf optimization [7], artificial hummingbird algorithm [8], cuckoo search [9], squirrel search algorithm [10], genetic algorithm [11], ant colony optimization [12], bat algorithm [13], glowworm swarm optimization [14], fruit fly optimization [15], invasive weed optimization [16], flower pollination algorithm [17], and dolphin echolocation algorithm [18]). The ensuing interpretations and implications establish the GAHA-SVM model as an innovative and effective tool for detecting power theft. It further advances the field of metaheuristic optimization by addressing pertinent research gaps.

The study's major contributions are as follows:

- Firstly, comprehensive data preprocessing is done on the original dataset. This involves removing rows with missing values and replacing outliers with median values using *z*-score analysis.
- Additionally, the downsampling method addresses the dataset's class imbalance ensuring an equal distribution of fraudulent and nonfraudulent instances. Secondly, the preprocessed data get split into training, validation, and testing sets.
- Thirdly, normalization is carried out using mean and standard deviation values obtained exclusively from the training set, thus preventing any investigation of the test data before model evaluation.
- Fourthly, the GAHA-SVM algorithm performed the optimization within the training set to identify the best C and gamma hyperparameters for training the SVM model, with the data validation ensuring the model is generalized well without overfitting.
- Finally, the model is evaluated on a separate testing set using six evaluation metrics: accuracy, precision, *f*_score, recall, *g*_mean, and MCC, to assess its performance.

The paper is organized as follows: The literature review section discusses previous research on optimization algorithms, identifies gaps in current knowledge, and establishes the context for the study. The methodology section outlines the methods used to address the research questions and develop solutions. The results section presents the research findings, supported by figures and tables. The discussion sections analyze and interpret the research findings in the context of previous literature, and the study is summarized in the conclusion section.

## 2. Literature Review

Support vector machines (SVMs) are a widely used supervised learning algorithm, showing immense success in various fields. SVMs create decision boundaries to distinguish between classes, but overfitting can lead to poor performance on new or unseen data and require their hyperparameter tuning before training [19]. Metaheuristic algorithms, a class of optimization algorithms helpful in solving complex problems where traditional mathematical methods are not feasible due to their ability to balance exploitation and exploration [20], have emerged as powerful tools for optimizing support vector machines' hyperparameters. The optimal selection of machine learning algorithms' hyperparameters is crucial to achieving high modeling accuracy. In [21], Ali et al. noted that the large size of the problem space made fine-tuning hyperparameters a computational challenge asserting that traditional random search or grid search optimization techniques lead to slow convergence or long execution time at times. They tried several algorithms as an alternative to the grid search and reported GA as the most effective since it had a lower temporary time-consuming complexity than other algorithms. The authors of [22] showed that using GA to select SVM's radial basis kernel's optimal regularisation parameter (C) and cost factor gamma was profitable as machine learning hyperparameters' manual grid search tuning is very time-consuming. They further demonstrated that an SVM model with hyperparameters tuned by GA gave superior classification performance on face recognition models in contrast to the linear SVM in most data splits.

### 2.1. Review of Artificial Hummingbird Algorithm (AHA) Needs for Hybridization

The artificial hummingbird algorithm (AHA) is an optimization algorithm developed by Zhao et al. [8] that mimics the intelligent behavior of hummingbirds. It utilizes visit tables, hummingbirds, and food sources as significant components. Visit tables store different hummingbirds' visitor levels for each food source and show the time since a particular hummingbird's last visit. The most visited food sources for a specific hummingbird have an extra preference for gleaning more nectar. Hummingbirds use visit tables to arrive at their food source, updating these tables at every iteration.  Algorithm 1 shows the general structure of AHA [8].

Hummingbirds are first positioned at *n* food sources before being initialized randomly by the following equation:

(1) $$\begin{array}{c} x_{i}=\text{Low}+r\cdot \left(\text{Up}-\text{Low}\right)\;i=1,\ldots \ldots ,n. \end{array}$$

Low is a d-dimensional problem's lower bound, Up is its upper bound, *r* is a randomized vector [0, 1], *x*_*i*_ represents the *i*th food source position (the problem's solution), *i* is the food source index, and *n* is the population size.

Equation (2) gives the visit table initialization:

(2) $$\begin{array}{c} {\text{VT}}_{ij}=\left\{\begin{array}{cc} 0, & \text{if }i\neq j,\\ \text{null}, & i=j, \end{array}\right.\;i=1,\ldots ,n,\;j=1,\ldots ,n, \end{array}$$

where *i* = *j*, VT_*ij*_ = null means the hummingbird is feeding at its given food source while *i* ≠ *j*, and VT_*ij*_ = 0 shows that the *i*th hummingbird had just visited the *j*th food source in the current iteration.

Hummingbirds use guided foraging to find the most visited food sources and select the best nectar refilling rate. They use axial, radial, and omnidirectional flight skills to reach the food source. A directional switch vector controls the availability of directions in d-dimensional space. Equation (3) gives the axial flight in d-dimension space.

(3) $$\begin{array}{c} D^{\left(i\right)}=\left\{\begin{array}{cc} 1, & \text{if }i=\text{rand}i\left(\left[1,d\right]\right),\\ 0, & \text{else}, \end{array}\right.\;i=1,\ldots ,d, \end{array}$$

where *D*^(*i*)^ is the axial flight, randi ([1, *d*]) generates an integer randomly between 1 and *d*, *d* is the problem space dimension, and *i* represents the food source index.

Equation (4) gives the diagonal flight.

(4) $$\begin{array}{c} D^{\left(i\right)}=\left\{\begin{array}{cc} 1, & \text{if }i=P\left(j\right),\;j\in \left[1,k\right],P=\text{randperm},\\ 0, & \text{else}, \end{array}\right.\;P=\text{randperm}, \end{array}$$

where *D*^(*i*)^ is the diagonal flight, randperm(k) generates an integer permutation randomly between 1 and *k*, j is the dimension index, *k* is the number of elements to permute, *i* represents the food source index, and *P*(*j*) is the probability for the *j*^th^ dimension food source.

Equation (5) gives the omnidirectional flight.

(5) $$\begin{array}{c} D^{\left(i\right)}1\;i=1,\ldots d, \end{array}$$

where *D*^(*i*)^1 is the omnidirectional flight, *i* is the food source index, and *d* is the problem space dimension. These given flight skills mathematical models imitate the hummingbirds' searching behavior in multidimensional 3D space. Equations (6) and (7) provide the candidate's food source and guide foraging behavior.

(6) $$\begin{array}{c} V_{i}\left(t+1\right)=x_{i,\text{tar}}\left(t\right)+a.D.\left(x_{i}\left(t\right)-x_{i,\text{tar}}\left(t\right)\right), \end{array}$$

(7) $$\begin{array}{c} a∼N\left(0,1\right). \end{array}$$

In equation (6), *V*_*i*_(*t*+1) is the *i*^th^ food source at a time (*t* + 1), *x*_*i*_(*t*) is the food source at the *i*^th^ position during time *t*, *x*_*i*,tar_(*t*) denotes the food source that the *i*th hummingbird wants to fly to, *D* is the problem space dimension, and *t* is the current time step. In equation (7), a stands for the guided factor given as a normal distribution *N* (0, 1) where the mean is zero, the standard deviation is one, and *N* is the population size. The *i*th food source position gets updated by

(8) $$\begin{array}{c} x_{i}\left(t+1\right)=\left\{\begin{array}{cc} x_{i}\left(t\right), & f\left(x_{i\;}\left(t\right)\right)\leq f\left(\left(v_{i\;}\;\left(t+1\right)\right)\right.,\\ v_{i\;}\left(t+1\right), & f\left(x_{i\;}\;\left(t\right)\right)>f\left(\left(v_{i\;}\;\left(t+1\right)\right)\right., \end{array}\right. \end{array}$$

where *x*_*i*_(*t*+1) is the position of the *i*^th^ food source at the next step time (*t* + 1), *f* (.) is the function's fitness value, and *x*_*i*_ (*t*) and *v*_*i* _ (*t* + 1) remain the same as defined above in equation (6). Food sources are not changed during guided foraging unless the hummingbird finds a food source with a better nectar refill rate. Territorial foraging and the candidate food source are given mathematically by equations (9) and (10).

(9) $$\begin{array}{c} v_{i}\left(t+1\right)=x_{i}\left(t\right)+b.D.x_{i}\left(t\right), \end{array}$$

(10) $$\begin{array}{c} b∼N\left(0,1\right). \end{array}$$

In equations (9) and (10), *b* represents the territorial factor, its normal distribution is *N* (0, 1), where the standard deviation is one, and the mean is zero. *X*_*i*_(*t*) and *v*_*i*_(*t*+1) and *N* remain the same as defined in equations (6) and (7), respectively. After the territorial foraging strategy, the visit tables get updated.

The migratory foraging strategy in AHA involves a migration coefficient that enables hummingbirds to randomly migrate to new food sources when their nectar refilled randomly slows. Equation (11) represents the randomly produced migratory foraging strategy.

(11) $$\begin{array}{c} x_{\text{wor}}\left(t+1\right)=\text{Low}+r\cdot \left(\text{Up}-\text{Low}\right). \end{array}$$

In equation (11), *x*_wor_ represents the food source whose nectar takes the longest to refill, Low, Up, and *r* remain the same as defined in equation (1), and (*t* + 1) is the next step time after time (*t*).

Migratory foraging increases exploration and reduces the change of convergence into local optima. Higher exploitation occurs when an updated new food source drives hummingbirds to fly towards it. In the worst-case scenario, a hummingbird might visit a new target food source after 2*n* iterations, requiring the migration foraging strategy to explore the search space and improve stagnation. Equation (12) gives the migration coefficient relative to the population size.

(12) $$\begin{array}{c} M=2n, \end{array}$$

where *M* is the migration coefficient relative to the population size and *n* remains the same as given in equation (1)

In [8], Zhao et al. used two benchmark functions to test the AHA optimization performance and suggested the hybridization of AHA with stochastic operators or other optimization methods' search components to develop a better version for tackling binary or multiobjective problems. Many works have been done focusing on improving the exploration of AHA. The authors of [23] specified that although the artificial hummingbird algorithm has the advantages of a simplistic structure, ease of implementation, and speed in finding the global optimum solution, it faces limitations such as low convergence accuracy and getting stuck in the local optima. Their proposed hybrid-artificial hummingbird algorithm which incorporated PSO and Cauchy mutation to increase population diversity, improve accuracy and convergence, and prevent the AHA from being stuck in the local optimum was used to optimize complex shape-adjustable generalized cubic Ball (CSGC-Ball) curves and show competitive results and practicality in comparison to other advanced algorithms. In [24], the authors moved by AHA's imbalance in exploration and exploitation, premature convergence, and low precision introduced a hybrid artificial hummingbird algorithm called LCAHA which utilized Levy flight to introduce population diversity and prevent premature convergence. Their hybrid algorithm was tested on six engineering optimization cases and the 23 classical test suites where it demonstrated promising results and potential for solving practical applications. In [25], Abd Elaziz et al. introduced an enhanced version of AHA using a quantum-based optimization to improve its exploration ability. It showed good accuracy on their social IoT data but suffered time complexity. Alhumade et al. noted in [26] that AHA uses the visit table as an update to balance exploitation and exploration, efficiently exploring new regions while promisingly exploiting local solutions. However, AHA's exploration is not as robust as other algorithms causing it to get stuck in the local optima at times and thus return suboptimal solution.

### 2.2. Review of Machine Learning Classifiers and Metaheuristic Optimization Techniques for Various Applications

The accuracy of different machine learning models in identifying power theft was assessed in [27]. At 81%, the SVM model was the most accurate, followed by Naive Bayes (68%), K-nearest neighbors (79%), random forest (80%), and logistic regression (69%). The study also noted improvements in accuracy ranging from 3.8% to 11% compared to similar studies when comparing these results with earlier research. Nevertheless, because of inadequate data, the authors recognized that they could not accurately classify about 25% of cases of electricity theft.

Similarly, machine learning classifiers such as decision tree, SVM, Naive Bayes, and random forest were evaluated for accuracy [28]. With an accuracy of 72.5%, the decision tree model outperformed SVM, which came in last at 65.2%. Notably, after correcting for missing values through data preprocessing, the decision tree accuracy increased to 91.3%. It was deduced that decision trees perform better than other classifiers when data preparation was used.

Mia et al. [29] concentrated on using feature engineering and ensemble classification approaches to improve the detection of power theft. Several machine learning classifiers, such as KNN, random forest, gradient boosting, XGBoost, AdaBoost, and logistic regression, were assessed in the study. The most accurate model was random forest, with a 94.03% accuracy that rose to 97.06% via feature engineering. With random forest coming out on top, the authors demonstrated how feature engineering can be used to improve classifier performance.

Iftikhar et al. [30] presented a hybrid system integrating multilayer perceptron (MLP) with gated recurrent units (GRUs) to identify electricity theft in “Electricity Theft Detection in Smart Grid Using Machine Learning.” The study highlighted the difficulties caused by high false positive rates and unequal data distribution. The hybrid system outperformed earlier approaches assessed in the literature in terms of accuracy, precision, and area under the curve. The MLP-GRU model outperformed benchmark models (AlexNet, BGRU, and RNN) across various training and testing ratios, reaching a maximum training accuracy of 86% and a testing accuracy of 88% after 15 rounds. Except for AlexNet, it displayed faster execution times than the other benchmarks. The Diebold-Mariano test proved this model's great superiority over other models.

Salb et al. [31] developed a hybrid model combining CNN for feature extraction with XGBoost for intrusion detection. The model achieved 87.94% accuracy in multiclass classification, outperforming other methods. However, it struggled with binary categorization, requiring further tuning. The study highlights the model's adaptability to evolving risks and low maintenance costs but also acknowledges limitations in the optimization method and dataset scope.

In [32], Hassaballah et al. introduced an automated arrhythmia classification approach using metaheuristic optimization to enhance ECG analysis. The study achieved exceptional accuracy of 99.92% and sensitivity of 99.81% with SVM, KNN, GBDT, and RF classifiers. Experiments on three datasets demonstrated significant improvements, surpassing existing approaches. Further validation across diverse datasets and improved optimization methodologies are recognized as necessary.

Todorovic et al. in [33] utilized an augmented sine-cosine algorithm (SCA) to optimize hyperparameters for the XGBoost model used to predict audit opinions. The study used a dataset of 12,690 observations from Serbian companies, inclusive of existing and new clients. The improved model outperformed previous benchmarks, and SHAP value analysis provided insights into variable impacts, demonstrating the efficacy of combining metaheuristics with machine learning for audit opinion prediction.

In a study, published in the “Handbook of Whale Optimization Algorithm: Variants, Improvements, Hybrids, and Applications,” Oladayo Oladejo et al. [34] studied the use of the whale optimization algorithm (WOA) and its modifications to tune SVM hyperparameters using seven datasets. SVMs tuned using LWOA and WOAmM obtained the best classification accuracy, whereas WOA and MSWOA required the lowest tuning durations. This study demonstrates the usefulness of metaheuristics in balancing accuracy and computing economy for SVM hyperparameter adjustment.

Unlu [35] employed a hybrid model that included support vector machine (SVM) and Bayesian optimization (BO) to forecast credit card client attrition. The study tested four kernels (linear, polynomial, radial basis, and sigmoid) and showed that the linear kernel performed best, with an accuracy of 91%. The sigmoid kernel has the lowest accuracy, 84%. While BO successfully enhanced SVM hyperparameters, the study recommends conducting more research to evaluate alternative hyperparameter optimization methods and investigate other machine learning and deep learning algorithms for better churn prediction.

Abbas et al. [36] performed a comparison of hyperparameter optimization strategies for machine learning models utilized in landslide susceptibility mapping. The study focused on metaheuristic and Bayesian approaches, emphasizing their performance advantages over baseline algorithms. Metaheuristic algorithms boosted random forest model performance by up to 5% over grid search and random search approaches, while Bayesian optimization increased SVM accuracy by 6%. The study proposes that future research should investigate similar strategies in larger geographical contexts to improve model robustness and predictive capabilities.

The authors in [37] investigated metaheuristic optimization of deep learning (DL) models in energy load forecasting. After evaluating six algorithms, including the firefly algorithm (FA), they discovered that FA outperformed grid search with an *R*^2^ of 0.954082. This work emphasizes metaheuristics' usefulness in enhancing DL model correctness and calls for more research into advanced DL architectures and optimization methodologies.

Dobrojevic et al. [38] presented a unique CNN-ELM-HASCA hybrid model for IoT intrusion detection, which employs a modified sine-cosine algorithm (SCA) for parameter tuning. This technique outperforms seven previous metaheuristic algorithms examined, with classification accuracy of 98.67% and 96.65% on Windows 7 and Windows 10 IoT datasets, respectively. They proposed automating CNN structure optimization, verifying the model on various datasets, and investigating hybrid metaheuristic methodologies to improve IoT security solutions in future studies.

### 2.3. Review of Competitor Algorithms as Optimizers

Bio-inspired algorithms, which mimic living organisms' biological activities, have gained momentum in solving engineering problems [39–41]. Despite the multiplicity of works in that domain, the no free lunch optimization theorem states that there will never be an algorithm to solve all optimization problems effectively [5].

Genetic algorithm (GA) solves complex optimization problems by mimicking selection, crossover, and mutation behaviors. It initializes a population of individuals for each candidate solution, which evolves with these operators over time. The best individuals are used in the generation of a new population. However, it is prone to early convergence, and optimum performance depends on selected crossover rates, mutation, and objective function selection [5, 11]. The need to best utilize GA in hybridized algorithms to cover their inherent weakness has been covered in many studies. In their research, the authors of [42] emphasized that GA's successful implementation greatly hinges on its crossover and mutation operators since they reduce premature convergence. They further noted the computational efficiency of genetic algorithms as an area needing improvement. In [43], the authors extolled the virtues of GA as an adaptive technique used to solve complex problems and endorsed it for solving hybrid computational challenges. In [44], the authors noted that in certain situations, genetic algorithms are overkill, and to make them more efficient and effective, they should be hybridized with other techniques, and they made calls for future research to focus on the development of hybridized models to enhance efficiency.

The glowworm swarm optimization (GSO) is a swarm optimization algorithm that mimics the glow behavior of glowworms. It is competent in capturing the objective function's local optimum in infinite dimensional vector space; however, its reliance on a fixed step size, with the standard suggested fixed step size being 0.03, leads to a trade-off between convergence speed and accuracy [14].

Fruit fly optimization (FFO) is useful for difficult optimization problems and is modeled on how the fruit fly searches for food. Although fast, simply structured, and easy to implement, FFO's minimal accuracy in converging to the minimal point 0 in the single-peak function (*F*1) as well as slow convergence speed across the single dimensional multipeak functions (*F*2 and *F*3) and the multidimensional single-peak functions (*F*4 to *F*6) when contrasted with the adaptive FFO shows that FFO does get trapped in the local optima and struggle to efficiently find the global optimum [15].

Particle swarm optimization (PSO) is a well-known bio-inspired method that copies flocking birds' social behavior. The tendency of PSO to get stuck in the local optima in high-dimensional space and its low convergence rate were highlighted by the PSO's poor search accuracy and convergence performance on the Rosenbrock and Griewank functions. A new directed weight complex network PSO (DWCNPSO algorithm) was proposed to address these shortcomings [6].

Cuckoo search (CS) models the cuckoo's reproduction and Levy flight behavior. When compared to a variable length cuckoo search (VLCS) which incorporates variable length solution representations, the original CS algorithm showed a 20% slower convergence speed and a 15% larger distance to the global optimum. The quantitative improvements on the CS by the VLCS show the original CS's poor exploitation and low precision when converging [9].

Bat algorithm (BA) is modeled on micro-bat echolocation behavior and is useful for solving constrained and unconstrained optimization problems. However, the standard BA gets trapped in the local optima and also yields unstable results. An improved bat algorithm (IBA) algorithm is proposed in the study and it showed around 30% improvement in convergence speed and 25% reduction in optimization instability [13].

The squirrel search algorithm (SSA) is based on the foraging behavior of squirrels. Although its search space exploration is effective for multidimensional optimization problems, it exhibits decreased accuracy and slower convergence rates with higher dimensions from 30 to 100. Its performance diminished in reaching optimal solutions particularly when compared with the improved squirrel search algorithm (ISSA) and other competitors across various benchmark functions [10].

Dolphin echolocation algorithm (DEA) is a bio-inspired algorithm inspired by the echolocation behavior of dolphins. It has few parameters to set and gives excellent results with low computational efforts but needs balance in its exploration and exploitation. In [18], the authors demonstrated DEA's inferior exploration and exploitation by modeling an improved dolphin echolocation algorithm (IDEA) with SVM, KNN, and NB. This improved algorithm bested the normal DEA-SVM in all classification metrics across various ranges of cancer tweet datasets. For instance, considering 5000 cancer tweet datasets, IDEA-SVM accuracy, precision, recall, and *f*_score of 96.58%, 99.12%, 96.54%, and 97.5% outperformed their corresponding DEA-based classifiers.

Ant colony optimization (ACO) is an algorithm inspired by real ants' foraging behavior, used to approximate solutions to complex problems. It uses pheromone trails to find the shortest route between ants' nests and food sources on a weighted graph. However as shown by the authors in [12], traditional ACOs suffer from low efficiency and convergence issues. In the path planning task, they exhibited stagnation and a slow convergence rate, reaching only 50% of the optimal solution within 100 iterations.

The flower pollination algorithm (FPA) is an efficient metaheuristic inspired by the pollination of flowering plants. It is simple to formulate, gives high computational performance, and has cross-domain applicability, but its optimal parameters depend significantly on the objective functions. The problem dimensions and affordable computational cost and the ones that minimize mean prediction error do not always offer the most robust predictions. The research showed that optimum population size, scale factor, and switch probability should be between 20 and 40, 0.1 and 1.0, and 0.2 and 0.4, respectively [17].

The grey wolf optimization (GWO) algorithm is modeled after the grey wolf pack hierarchy and hunting behavior. The authors in [7] highlighted that traditional GWO faces instability and convergence issues and offered an improved GWO based on an improved dynamic weighing strategy for mobile robot path planning. Across 15 benchmark functions, the results show that the improved GWO offers better solution speed and accuracy.

Invasive weed optimization (IWO) is a population-based evolutionary optimization method inspired by weed colonies. While it has good exploration, its poor exploitation ability causes it to get trapped in the local optimum and converge prematurely when solving complex problems. The authors in [16] offered an improved chaotic IWO to set the optimal parameter of the PID controller and compared the results with the normal IWO. The improved version gives a better settling time of 0.690 seconds to 0.344 seconds, a better cost function of 8.0369 to 23.0944, and better convergence than the ordinary one.

In summary, despite having a wide range of applications, general bio-inspired algorithms face one or more issues such as an imbalance in exploration and exploitation, getting trapped in the local optimum, premature convergence, poor local search ability, or computational expense necessitating improved versions.

## 3. Proposed Genetic Artificial Hummingbird Algorithm-Support Vector Machine

Segera et al. noted in [45] that exploration and exploitation are antagonistic principles that require balancing for metaheuristics to improve performance. They suggested the development of a memetic algorithm whereby an integration of two or more algorithms is done to enhance overall performance. The authors of [46] noted that swarm-based algorithms' success is highly dependent on the balance between exploitation and exploration. In this light, a genetic artificial hummingbird algorithm (GAHA) is proposed for optimizing the support vector machine to perform the classification of electricity users.

### 3.1. GAHA-SVM Optimization vs. SVM's Grid Search

Traditionally, grid search has been a popular method for hyperparameter tuning, where a predefined grid of hyperparameter values is exhaustively searched to find the best combination. Using the RBF function, the SVM performs the grid search by evaluating the model's performance over different combinations of C and gamma from the predefined grid range of *C* = [2^−5^, 2^3^] and gamma = [2^−2^, 2^3^]. Stratified hold-out cross-validation done in an 80-10-10 (training, validation, and testing) ratio prevents the model from overfitting and ensures it can generalize on unseen data. Performance metrics such as accuracy, precision, *f*_score, recall, *g*_mean, and MCC are calculated for each combination of *C* and gamma values in the grid and compared across every hyperparameter configuration to identify the combination yielding the highest performance. Once the optimal hyperparameters from that process are identified, the final model is trained, its performance is evaluated on the independent test set, and the evaluation metrics are computed.

In contrast, GAHA-SVM uses a hybrid method combining the genetic algorithm's mutation and crossover operators with the artificial hummingbird migratory foraging stage to efficiently search for the optimal C and gamma hyperparameters. Hyperparameters are initiated over the range of *C* = [2^−5^, 2^3^] and gamma = [2^−2^, 2^3^]. The population of 20 hummingbirds is iteratively evolved based on the population's fitness. The algorithm uses the hummingbird's guided foraging when a randomly generated number (rand) is less than 0.5, territorial foraging when it is greater than 0.5, and migratory foraging after every four iterations to search the search space. Radial flight is utilized if the random number is less than 1/3 and omnidirectional flight is utilized if it is greater than 2/3, and axial flight is used otherwise. During migratory foraging, the best-performing individual in the population undergoes genetic algorithm operations (mutation and crossover) to generate new offspring solutions bringing diversity to the population and allowing for exploration of new regions in the search space. Each solution fitness is evaluated using the GAHA-SVM's model performance on the validation set and the best solution is updated if a better one is found. If, after fifteen iterations, the difference between the current best fitness and the best fitness fifteen iterations ago is less than the best fitness error of 1*e* − 10, the loop can be broken, and the algorithm is considered to have converged. If not, it continues for the specified number of iterations (100). This hybrid approach ensures adaptive exploration and exploitation of the hyperparameter space leading to improved convergence. The optimal C and gamma hyperparameters selected from this process are used to predict a separate testing set and then compute the evaluation metrics.

The GAHA-SVM search is more advantageous when compared to the SVM's traditional grid search. The iterative adjustments based on fitness values allow for a more efficient exploration and exploitation of the search space when contrasted with grid search which relies on a fixed grid of hyperparameter values. Next, the usage of the genetic mutation and crossover operators during the GAHA search promotes population diversity and prevents premature convergence to suboptimal solutions. For these reasons, the GAHA-SVM search effectively converges and produces superior optimal C and gamma hyperparameters when contrasted with the grid search.

### 3.2. Improvement on Bio-Inspired Optimizers' Shortcomings

Hybridizing the artificial hummingbird algorithm with genetic algorithm's mutation and crossover operators can lead to a more versatile and efficient optimizer with improved performance and the potential to address the most common pitfalls of bio-inspired optimizers, as outlined below.

- *Imbalance in Exploration and Exploitation*. The artificial hummingbird algorithm is known for its strong exploitation capabilities, while the genetic algorithm mutation and crossover operators excel in exploration. Combining the two can better balance exploration (diversity) and exploitation (exploiting promising regions). The GA introduces randomness and diversity into the artificial hummingbird algorithm, enhancing its exploration capabilities.
- *Getting Trapped in the Local Optimum*. Genetic Algorithms are effective at escaping local optima due to their use of the mutation and crossover operators. By incorporating these operators into the artificial hummingbird algorithm, random perturbations are introduced to allow the algorithm to explore different regions of the search space.
- *Converging Prematurely*. Premature convergence occurs when an algorithm stops exploring the search space too early and settles for suboptimal solutions. Diversity is introduced by integrating GA mutation and crossover operators, and premature convergence is avoided.
- *Poor Local Search*. Genetic algorithms are not known for their strong local search capabilities. However, by combining them with the artificial hummingbird algorithm, which has better local search abilities, we can leverage the strengths of both algorithms. The hummingbird algorithm can perform local search around promising solutions, while the mutation operators from the genetic algorithm can provide additional exploration and diversification.

### 3.3. Modeling of the Genetic Artificial Hummingbird Algorithm-Support Vector Machine (GAHA-SVM) Classifier

The algorithm begins by initializing various parameters, such as the maximum number of iterations (MaxIt), hummingbird population size (nPop), and the training and testing data (XTrain, YTrain, XTest, and YTest). The fitness of each hummingbird is evaluated using the Benchmark_SVM function, which corresponds to the SVM classification accuracy with specific C and gamma values. Next, the algorithm enters the main loop (It = 1: MaxIt), where the hummingbirds perform different kinds of flights (diagonal, omnidirectional, or axial) based on random probabilities. After every nPop/5 iteration, the algorithm triggers migration foraging, where the best-performing hummingbird migrates and is replaced by offspring generated through the GA's operations with the visit table updated accordingly. The roulette wheel selections set the probability of an individual being selected to be directly proportional to their fitness compared to the rest of the population. It is given mathematically by

(13) $$\begin{array}{c} S_{i}=\frac{f\left(i\right)}{\sum_{i=1}f\left(i\right)}\times 100\%, \end{array}$$

where *S*_*i* _ is the selected individual, *i* is the chromosome, and *f*(*i*) is the fitness of the *i*^th^ chromosome.

Whether crossover occurs is decided by

(14) $$\begin{array}{c} c=\left\{\begin{array}{cc} \text{Co}\left(m,\;\pi \right), & \text{if }\left(r<P\right),\\ m, & \text{otherwise}, \end{array}\right. \end{array}$$

where *P* is the crossover probability, *c* is the crossover individual, *m* is the mutated individual, *π* is the best-performing hummingbird from the previous generation, *r* [0, 1] is a uniformed random number, and Co is the crossover operation. The crossover operation gets initiated when the randomly produced number is less than the crossover probability, *r* < *P*, to produce the crossover individual (c).

The formula for the mutation operation is given by

(15) $$\begin{array}{c} m=\left\{\begin{array}{cc} \text{insert}\left(\pi \right), & \text{if }\left(r<Pm\right),\\ \text{swap}\left(\pi \right), & \text{otherwise}, \end{array}\right. \end{array}$$

where Pm is the mutation probability, and the definition of *m*, *r*, and *π* remains the same as defined in equation (14). If the randomly created number is less than the mutation probability, we retain the previous best-performing hummingbird. Otherwise, we swap that hummingbird with the mutated individual. The algorithm's best fitness and its corresponding solutions are updated accordingly.  Algorithm 2 summarizes the genetic operation during the GAHA-SVM migratory foraging stage.

The algorithm checks the difference between the last fifteen best fitness values and the current best fitness value using equation (16). If the difference is less than the best fitness error, it breaks the loop and returns the best solutions BestX_C and BestX_gamma.

(16) $$\begin{array}{c} \text{If It}>15\left(\text{HisBestFit}\left(\text{It}-15\right)-\text{HisBestFit}\left(\text{It}\right)\right)<\text{error BF break}; \end{array}$$

where It is the iteration, HisBestFit is the history of the best fitness, and error_BF is a very small value required to terminate the iterations.

The complexity of the proposed GAHA in terms of fitness function evaluations is given as follows:

(1) Initialization and data preparation:
  - Similar to AHA and GA, GAHA involves data loading, sampling, and feature separation: O (N).
  - Cross-validation partitioning: O (N).
(2) Normalization:
  - Feature normalization: O (N. d).
(3) Initialization of GAHA-SVM:
  - Hummingbird's population and parameters initialization: O (P. d).
  - Initial fitness evaluation using Benchmark_SVM: O (P. d (N^3^)).
(4) Main GAHA loop:
  - Hummingbird's movement and direction vector computation: O (P. d).
  - Global and local exploration: O (P. d (N^3^)).
(5) Elitism and replacement:
  - Similar to GA, there are population replacement and elitism: O (P. d).
(6) Convergence and termination:
  - Termination condition and convergence criteria: O (MaxIter).
  - Final evaluation and metric computation: O (d).

GAHA's overall complexity per iteration is thus summarized in the following equation:

(17) $$\begin{array}{c} O\left(\text{MaxIter}.\;P.d.\left(N^{3}+d\right)\right). \end{array}$$

MaxIter is the maximum number of iterations, *P* is the population size, *d* is the hyperparameter space dimensionality, *N* is the SVM evaluation complexity, and *O* is the algorithm's upper bound.

The basic GA evaluates fitness using a straightforward evolutionary approach that depends on population size and generations and is given by

(18) $$\begin{array}{c} O\left(\text{Generations}.\;P.\;\left(N^{3}+d\right)\right), \end{array}$$

where Generations represent the number of iterations the GA runs, *P* is the population size, *d* is the hyperparameter space dimensionality, *N* is the SVM evaluation complexity, and *O* is the algorithm's complexity upper bound.

Basic AHA evaluates fitness similar to GAHA using hummingbird foraging behaviors and memory updates and is dependent on population size, iteration number, and dimensionality. It is given by

(19) $$\begin{array}{c} O\left(\text{MaxIter}.\;P.d.\;\left(N^{3}+d\right)\right), \end{array}$$

where MaxIter is the maximum number of iterations, *P* is the population size, *d* is the hyperparameter space dimensionality, *N* is the SVM evaluation complexity, and *O* is the algorithm's complexity upper bound.

GAHA combines the benefits of AHA and GA leading to better optimization results but at higher computational cost. Though GAHA and AHA share a similar complexity structure, GAHA is possibly more complex due to the additional GA phase, while GA is less complex in terms of FFEs compared to both.

GAHA offers novelty in comparison to the original AHA in the ways outlined below:

- *Frequency of Migratory Foraging Interval*. GAHA offers an alternate approach to migratory foraging by altering the exploration frequency from 2n to n/5, a 95% increase in exploration frequency, where *n* is the population size. This altercation ensures efficient exploration, thereby helping address the limitation of nonrobust exploration in the traditional AHA.
- *Hybrid Genetic Mechanisms*. GAHA incorporates genetic mutation and crossover techniques during migratory foraging, enhancing the algorithm's exploration capabilities. The combination of guided, territorial, and genetically informed migratory foraging allows for a more diverse exploration of the solution space, potentially leading to improved convergence and solution quality in contrast to AHA's reliance solely on traditional foraging strategies.
- *Dynamic Solution Replacement*. In GAHA, the replacement of solutions during the migratory foraging involves either the crossover or mutated individual replacing the worse solution. This dynamic solution replacement mechanism introduces an element of flexibility and adaptability. It allows the algorithm to leverage the strengths of genetic operators and AHA's traditional foraging strategies. This results in improved convergence and refined solutions. The traditional AHA, on the contrary, keeps recycling old solutions.

By extension, GAHA also varies from the competitor algorithms in this study in the following way:

1. *Adaptive Migratory Foraging with Genetic Elements*. Although GA utilizes genetic operators, GAHA specifically adapts the migratory foraging intervals and employs genetic mechanisms during the exploration, offering a hybrid approach. The rest of the competitor algorithms do their exploration without the incorporation of genetic mechanisms.
2. *Dynamic Solution Replacement with Stochastic Processes*. GAHA introduces stochastic processes during dynamic solution replacement, combining genetic operators with randomness for improved adaptability and exploration while the competitor algorithms, GA aside, use a deterministic replacement strategy during exploration.

**Figure 1:**
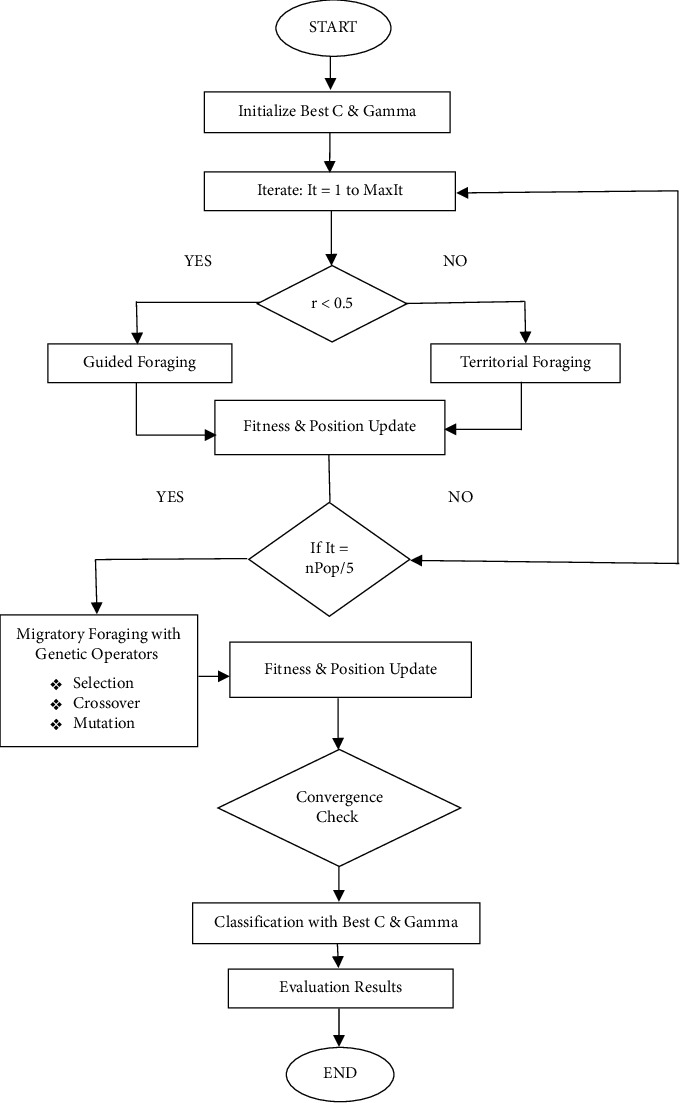
GAHA-SVM flowchart.

In summary, GAHA's novelty lies in its altered migratory foraging intervals, the integration of genetic mechanisms during exploration, dynamic solution replacement with stochastic processes, and combining the AHA traditional foraging strategies with genetic elements. These characteristics distinguish it from other optimization algorithms and give it a better chance of finding the best optimal solutions. The pseudocode for the GAHA-SVM model is given by Algorithm 3 and its flowchart is shown in Figure 1.

## 4. Methodology

Simulations were done in MATLAB R2023a software using a Lenovo IdeaPad Slim 7 Pro I4IHUS computer with Intel (R) Core™ i7-11370H @3.30 GHz. It has an installed RAM of 16 GB and a ROM of 952 GB running on a 64 bit operating system, x64-based processor. In addition to its ease of learning and user-friendly interface, MATLAB provides strong visualization and plotting capabilities thanks to its nice toolbox for graphical works. Furthermore, it provides excellent support for optimization and numerical analysis problems thanks to its optimization toolbox.

### 4.1. Nature of the Data

This research examines historical electricity consumption data from the Liberia Electricity Corporation AMI meter for a year from June 30, 2021, to June 30, 2022, focusing on the Peace Island Community in Monrovia, Liberia. The data include 60,666 rows and 13 columns, including ID_No, date, time, Global_Active_Power, Global_Reactive_Power, Power_Factor, Global_Intensity, Voltage, Billing, Sub_metering_1, Sub_metering_2, Sub_metering_3, and Status. The data are skewed, with 42,466 instances of fraud and 18,200 instances of nonfraudulent cases, representing 70 and 30 percent of the data, respectively, with the ground truth on fraudulent and nonfraudulent cases based on energy audit reports. The research focused on the two pertinent features: billing and global active power, as input. Billing represents the amount in USD billed to the customer's KWh consumption and global active power measures the actual power consumed in KWh. These features are selected based on their direct use in identifying anomalies that indicate electricity theft during energy audits. Status which tells whether the electricity usage is fraudulent or nonfraudulent is the output parameter that the model aims to predict.  Table 1 gives a snippet of the data fragment showing these input and output parameters.

### 4.2. Data Preprocessing

Data preprocessing is crucial for improving classification models due to noise, missing values, and outliers. The research performs preprocessing which includes missing value removal, outliers' replacement and data balancing, and data normalization on the 80 percent training set after the data had been split into training, validation, and testing sets. Historical electricity data are input into MATLAB for preprocessing, and the output is returned as a mat file. Since the dataset is of ample size to draw reliable conclusions, instances with missing values are removed. Similarly, the majority class is downsampled to ensure an equal number of both instances. Downsampling which involves the random removal of majority class instances ensures that both classes are equally represented and model bias towards the majority class is mitigated. It is less prone to overfitting, compared to oversampling techniques that generate synthetic samples which can potentially introduce noise and redundant samples. Equations (20) and (21) perform outliers' replacement and Z-score normalization, respectively.

(20) $$\begin{array}{c} f\left(xi\right)=\left\{\begin{array}{cc} \text{avg}\left(x\right)+2\text{std}\left(x\right), & \text{if }xi>\text{avg}\left(x\right)+2\text{std}\left(x\right),\\ xi, & \text{else}, \end{array}\right. \end{array}$$

where std (*x*) was the standard deviation of *x*, avg (*x*) was x's average value, *xi* is a particular instance of *x*, and *f*(*xi*) is the new value in the case that outlier replacement was done.

(21) $$\begin{array}{c} Z=\frac{A-\text{mean}\left(A\right)}{\text{std}\left(A\right)}, \end{array}$$

where *Z* is the feature normalized value, *A* represents the dataset, std is the standard deviation, and the mean is the average.

*Z*-score normalization, also known as standardization, is a crucial preprocessing step in machine learning to ensure that the features have similar scales and distributions. The mean and standard deviation for the normalization are calculated solely from the training dataset preventing data leakage.

### 4.3. GAHA-SVM Classification

The GAHA-SVM classifier inputs the balanced data table created after the preprocessing step. It randomly selects 7,270 samples for the model. The selection is randomized to prevent bias and overfitting. This process is repeated 15 times to ensure there is no bias and the results are averaged at the end. Of these 7,270 samples, 80 percent is used to train the model, 10 percent is used for validation, and 10 percent is used for testing the model. The model utilizes a benchmark function for calculating the best fitness, a grid search function that defines the range over which the C and gamma hyperparameters are being searched, a prediction function for calculating the mean of the model's confusion matrix, the main function of the code, and the genetic algorithm function with its associated files. The optimization algorithm was initiated with a population size (nPop) of 20 hummingbirds and a maximum iteration of 100 over 15 dimensions (dim). The rand function randomly generates the position of each hummingbird in the search space. The RBF kernel function is used because of its ability to capture complex nonlinear relationships in the data. The hyperparameters' grid search range was set at *C* = [2^−5^, 2^3^] and gamma = [2^−2^, 2^3^] with the upper and lower bounds specified as (Low_C, Up_C) and (Low_gamma, Up_gamma), respectively. The visit table for keeping track of the number of times each hummingbird in the population visits a given position is initialized as NaN values. The best fitness (BestF) and the best solutions (BestX_C and BestX_gamma) were initialized as an empty array. The best fitness error (error_BF) required to terminate the iterations was set at 1*e* − 10. The algorithm updates the best fitness value and solutions every iteration. If, after fifteen iterations, the difference between the current best fitness and the best fitness fifteen iterations ago is less than the best fitness error, the loop can be broken, and the algorithm is considered to have converged. If not, it continues for the specified number of iterations. The algorithm returns the mean of the best hyperparameters, the best fitness, the history of the best fitness, and the visit table. The fitcsvm model uses the best C and gamma hyperparameters to perform classification based on the test data. The confusion matrix and the model's accuracy, precision, *f*_score, recall, MCC, and *g* scores are all returned.

### 4.4. Comparison with Other Classifiers

The assessment outcomes from the GAHA-SVM were compared to those of thirteen other classifier algorithms that were executed. Consistent with the GAHA-SVM's design, 7270 instances of the data were randomly selected and utilized for each model, with 80% of the data being used for model training 10% being reserved for validation, and another 10% for testing. This was repeated 15 times and the results were averaged to ensure there was no bias. The same convergence condition was set as follows: if after fifteen iterations, the difference between the current best fitness and the best fitness fifteen iterations ago is less than the best fitness error, the loop should be broken, and the algorithm is considered to have converged. The best solutions (BestX_C and BestX_gamma) over the 15 dimensions are returned and used by the fitcsvm model for classification. The model performs classification on the test data and returns the confusion matrix mean taken over the fifteen dimensions. It also returns the model's accuracy, precision, *f*1score, recall, MCC, and *g*_score and the history of the best fitness. The fitness function which assesses the GAHA and its competitors' classification accuracy on the validation set is given by Algorithm 4.

The objective function of the optimization aims to minimize the classification error rate of the SVM model. This is achieved by optimizing the hyperparameters C and gamma to achieve the highest accuracy on the validation set. This means minimizing the fitness values as given by equation (22) below:

(22) $$\begin{array}{c} \text{Fit}=1-\text{mean}\left(\text{accuracies}\right), \end{array}$$

where Fit is the fitness value representing the objective function being minimized, mean is the average accuracy of the SVM model on the test set, and accuracies represent an array representing the proportion of correct prediction made over each run over the test set.

The PSO-SVM algorithm was initiated with a population size (nPop) of 20 particles, a dimension (dim) of 15, and a Maximum Iteration (MaxIt) of 100. The hyperparameters' grid search range was set at *C* = [2^−5^, 2^3^] and gamma = [2^−2^, 2^3^] in the Grid Search function. The inertia weight (w) was set at 0.7, and the cognitive weights 1 and 2 were set at 0.2.

The AHA-SVM algorithm was initiated with a population size (nPop) of 20 hummingbirds, a dimension (dim) of 15, and a Maximum Iteration (MaxIt) of 100. The visit table for keeping track of the number of times each hummingbird in the population visits a given position is initialized as NaN values. The hyperparameters' grid search range was set at *C* = [2^−5^, 2^3^] and gamma = [2^−2^, 2^3^] in the Grid Search function.

The GWO-SVM algorithm was initiated with a population size (nPop) of 20 wolves, a dimension (dim) of 5, and a Maximum Iteration (MaxIt) of 100. The hyperparameters' grid search range was set at *C* = [2^−5^, 2^3^] and gamma = [2^−2^, 2^3^] in the Grid Search function. The random values for *r*1 and *r*2 were selected as *r*1 = *r*2 = rand (PopSize, Dim).

The DEA-SVM algorithm was initiated with a population size (nPop) of 20 dolphins, a dimension (dim) of 15, and a Maximum Iteration (MaxIt) of 100. The hyperparameters' grid search range was set at *C* = [2^−5^, 2^3^] and gamma = [2^−2^, 2^3^] in the Grid Search function. The search area was set at 0.5, and the detection range was set at 0.1.

The BA-SVM algorithm was initiated with a population size (nPop) of 20 bats, a dimension (dim) of 15, and a Maximum Iteration (MaxIt) of 100. The hyperparameters' grid search range was set at *C* = [2^−5^, 2^3^] and gamma = [2^−2^, 2^3^] in the Grid Search function. The loudness was set at 1, the pulse rate at 0.5, and both the loudness scaling factor and pulse rate scaling factor at 0.9.

The FFO-SVM algorithm was initiated with a population size (nPop) of 20 fruit flies, a dimension (dim) of 15, and a Maximum Iteration (MaxIt) of 100. The hyperparameters' grid search range was set at *C* = [2^−5^, 2^3^] and gamma = [2^−2^, 2^3^] in the Grid Search function.

The SSA-SVM main code was initiated with a population size (nPop) of 20 squirrels, a dimension (dim) of 15, and a Maximum Iteration (MaxIt) of 100. The hyperparameters' grid search range was set at *C* = [2^−5^, 2^3^] and gamma = [2^−2^, 2^3^] in the Grid Search function. The maximum gliding distance was set at 1.11, the minimum gliding distance at 0.5, and the gliding constant at 1.9.

The GSO-SVM algorithm was initiated with a population size (nPop) of 20 glowworms, a dimension (dim) of 15, and a Maximum Iteration (MaxIt) of 100. The hyperparameters' grid search range was set at *C* = [2^−5^, 2^3^] and gamma = [2^−2^, 2^3^] in the Grid Search function. The glowworms' sensing range was set at 3, and the detection range was set at 0.4.

The IWO-SVM algorithm was initiated with a population size (nPop) of 20 weeds, a dimension (dim) of 15, and a Maximum Iteration (MaxIt) of 100. The hyperparameters' grid search range was set at *C* = [2^−5^, 2^3^] and gamma = [2^−2^, 2^3^] in the Grid Search function. The weed's probability of reproduction was set at 0.8, with the possibility of elimination and dispersion set at 0.1.

The ACO-SVM main code was initiated with a population size (nPop) of 20 ants, a dimension (dim) of 15, and a Maximum Iteration (MaxIt) of 100. The hyperparameters' grid search range was set at *C* = [2^−5^, 2^3^] and gamma = [2^−2^, 2^3^] in the Grid Search function. The ant's pheromone importance factor was set at 1, the heuristic importance factor at 2, and the pheromone evaporation rate at 0.1.

The CS-SVM main code was initiated with a population size (nPop) of 20 cuckoos, a dimension (dim) of 15, and a Maximum Iteration (MaxIt) of 100. The hyperparameters' grid search range was set at *C* = [2^−5^, 2^3^] and gamma = [2^−2^, 2^3^] in the Grid Search function. The probability of egg discovery was set at 0.25, and the step size was set at 0.1.

The FPA-SVM main code was initiated with a population size (nPop) of 20 flowers, a dimension (dim) of 15, and a Maximum Iteration (MaxIt) of 100. The hyperparameters' grid search range was set at *C* = [2^−5^, 2^3^] and gamma = [2^−2^, 2^3^] in the Grid Search function. The flower's pollination probability was set at 0.8, the pollen dispersal coefficient at 0.5, the flower's competitive strength at 1, and the flower's attraction coefficient at 2.

The GA-SVM main code was initiated with a population size (nPop) of 20, a dimension (dim) of 15, and a Maximum Iteration (MaxIt) of 100. The hyperparameters' grid search range was set at *C* = [2^−5^, 2^3^] and gamma = [2^−2^, 2^3^] in the Grid Search function. The crossover rate was set at 0.8, the mutation rate at 0.1, and the number of elites at 2.

Finally, a normal SVM classification was done using a traditional grid search using the same data sample size. Over 15 independent runs, the preprocessed data are split into training, validation, and testing sets in an 80-10-10 ratio, and the training features are normalized. A grid search is performed over the range at *C* = [2^−5^, 2^3^] and gamma = [2^−2^, 2^3^] to obtain the optimal hyperparameter configuration for the SVM model which is then trained and evaluated using the testing set which is completely unseen during the optimization process. The RBF kernel function is used. The best hyperparameters obtained were then used to train the model and evaluation was done on the separate testing set. SVM's evaluation results were compared against GAHA-SVM's results. This comparison highlights the improvements that GAHA-SVM offers over SVM's traditional grid search method.  Table 2 gives a summary of the algorithm parameter settings.

### 4.5. Evaluation

The GAHA-SVM algorithm and its competitor algorithms' efficiency is evaluated using the following performance metrics: accuracy, precision, f_score, recall, MCC, and gscore. The model accuracy is calculated by equation (23). The accuracy tells which percent of the predictions were correct.

(23) $$\begin{array}{c} \text{Accuracy}=\frac{\left(\text{TP}+\text{TN}\right)}{\left(\text{TP}+\text{TN}+\text{FP}+\text{FN}\right)}, \end{array}$$

where TP is true positive, TN is true negative, FP is false positive, and FN is false negative. The precision is calculated by equation (24) and tells the number of true fraudulent users from the number of predicted fraudulent users by the classifier.

(24) $$\begin{array}{c} \text{Precision}=\frac{\left(\text{TP}\right)}{\left(\text{TP}+\text{FP}\right)}, \end{array}$$

where TP and FP remain the same as defined in equation (23). The f1score also known as the *f*_score is calculated by equation (25). It is a balanced measure of the model performance between precision and recall.

(25) $$\begin{array}{c} F1\text{ score}=\frac{\left(2\;x\text{ TP}\right)}{\left(2\;x\text{ TP}+\text{FP}+\text{FN}\right)}, \end{array}$$

where TP, FP, and FN are the same as defined in equation (23). The recall is calculated by equation (26). It measures the proportion of correctly predicted positive instances out of all positive instances.

(26) $$\begin{array}{c} \text{Recall}=\frac{\left(\text{TP}\right)}{\left(\text{TP}+\text{FN}\right)}. \end{array}$$

The MCC is calculated by equation (27). It is reliant on the confusion matrix (TP, TN, FP, FN) to have good results. Its scores range between −1 and 1.

(27) $$\begin{array}{c} \text{MCC}=\frac{\left(\text{ TP }x\text{ TN}-\text{FP }x\text{ FN}\right)}{\left(\sqrt{\;\left(\text{TP}+\text{FP}\right)\left(\text{TP}+\text{FN}\right)\left(\text{TN}+\text{FP}\right)\left(\text{TN}+\text{FN}\right)}\right.}, \end{array}$$

where TP, TN, FP, and FN remain the same as defined in equation (23). The geometric mean, known as the G-mean for short or sometimes the G-score, measures the balance between precision and recall and is useful when the dataset is imbalanced. It is given by equation (28) below.

(28) $$\begin{array}{c} G_{\text{mean}}=\sqrt{\text{precision }x\text{ recall}}, \end{array}$$

where precision and recall are as defined in equations (24) and (26), respectively.

### 4.6. Benchmark Test Functions

As is commonly done in the field of optimization and evolutionary computation, the proposed algorithm and its competitors were tested on the 23 standard unimodal, multimodal, and fixed-dimensional multimodal benchmark functions. Each of those three sets of benchmark functions evaluates the performance and robustness of the algorithm and its competitors in a specific way. The unimodal functions are functions with a single peak or global optimum. They show the algorithm's exploitation abilities and how well they converge to a single solution. The multimodal benchmark functions have multiple peaks or local optima. They show the algorithm's ability to explore the search space and find multiple solutions while avoiding getting stuck in the local optima. The fixed-dimensional multimodal functions are an extension of the multimodal concept of problems with fixed dimensionality. They evaluate the algorithm's convergence accuracy in scenarios where the dimensionality of the problem is fixed. The algorithms were tested with a population size of 20, with the initial population being randomly generated, and were run with a maximum of 500 iterations. The run is repeated 15 times for each benchmark function and the average and standard deviation of their minimum fitness are obtained as well as their average time.

### 4.7. Wilcoxon Rank-Sum Tests

The statistical analysis in this study employs the Wilcoxon rank-sum test. To compare the GAHA-SVM algorithm with 13 competitor metrics, each pair is evaluated separately using six evaluation metrics. The objective is to determine whether there exists a significant statistical difference between GAHA-SVM and its competitors in each paired comparison. A significance level of 5% is chosen for the Wilcoxon rank-sum tests. A *h* value of 1 indicates a significant statistical difference, whereas a *h* value of 0 suggests no significant statistical difference between the observed pairs. In addition, the *p* value was compared to the significance level of 5% to determine if the difference between the population median and the hypothesized was statistically significant. Where the *p* value was less than or equal to the significance level, it indicates that there is a significant statistical difference between the hypothesized median and population median, and the null hypothesis is rejected. On the contrary, when the *p* value is greater than the significance level, it is concluded that there is no significant statistical difference and the null hypothesis is accepted.

## 5. Results and Discussion

This section presents the results from implementing and evaluating the genetic artificial hummingbird algorithm-support vector machine (GAHA-SVM) classifier and its competitors using historical electricity consumption data.  Table 3 gives the data summary and Table 4 gives the effect of the data balancing. Results including evaluation metrics, benchmark tests, and Wilcoxon rank-sum tests are presented with the aid of graphs and figures, with interpretations and implications drawn from those results.

### 5.1. Evaluation, Benchmark Functions, and Statistical Test Results

**Figure 2:**
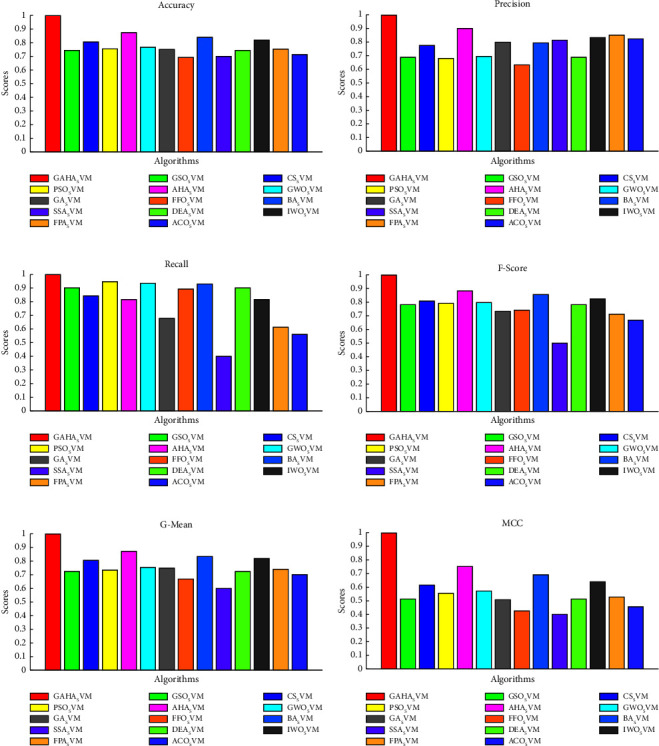
GAHA-SVM's evaluation metrics compared with 13 competitor algorithms.

**Figure 3:**
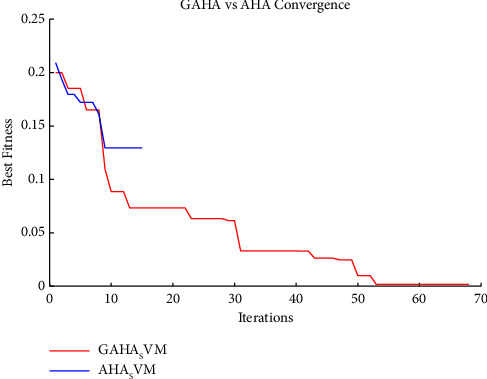
GAHA vs. AHA convergence curve.

**Figure 4:**
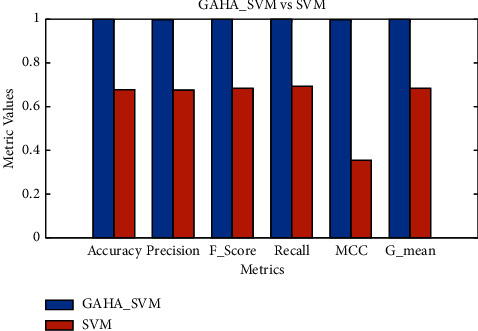
GAHA-SVM's evaluation metrics compared to SVM's grid search.

**Figure 5:**
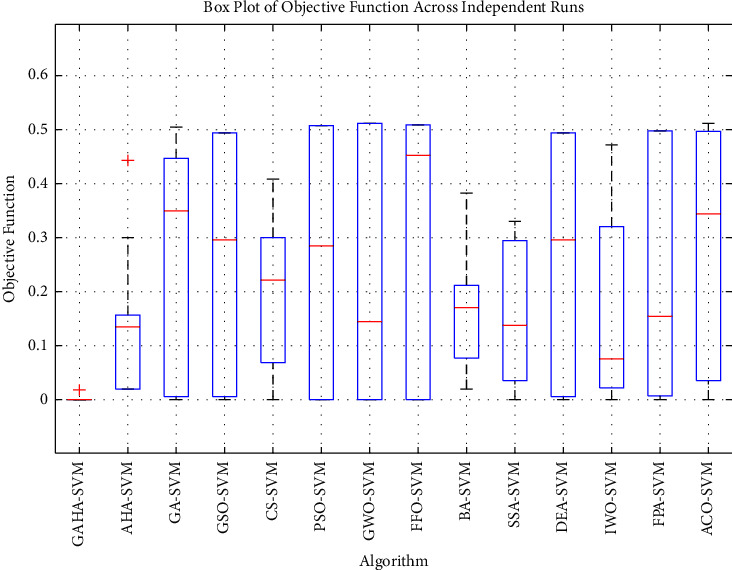
Box plot of GAHA-SVM and competitor algorithms over the 15 runs.

Using the preprocessed and balanced data, the GAHA-SVM hybrid algorithm was assessed against the research's predefined objective of power theft detection. The GAHA-SVM hybrid algorithm was utilized to detect power theft using preprocessed and balanced data. The algorithm was trained on 80% of the data over 15 independent runs, validated on 10%, and tested on the remaining 10%. The optimal C and gamma values of [5.7531, 0.279] were obtained and used by the fitcsvm function to classify users as fraudulent or nonfraudulent. The GAHA classifier returned a confusion matrix of [347 0, 1 379] indicating the model's [TP, FN; FP, FN]. The average time, best fit, and standard deviation were 4656.18 seconds, 0.0016, and 0.0550, respectively. The same process was repeated for 13 competitor algorithms, and the results are summarized in Table 5 to fulfill research objectives three and four. The GAHA-SVM algorithm's classification ability was evaluated using six evaluation metrics, returning accuracy, precision, *f*_score, recall, MCC, and *g*_mean of 0.9986, 0.9971, 0.9986, 1, 0.9972, and 0.9987, respectively. The 13 alternative algorithms were also evaluated, and their results were compared to the GAHA-SVM algorithm in Figure 2. In fulfillment of the second research objective, the convergence of the GAHA-SVM algorithm was compared to AHA-SVM in Figure 3, to observe the improvement made on the latter by the genetic operators. The normal SVM classifier was also used, and it returned a confusion matrix of [253 113, 122 239] indicating the model's [TP, FN; FP, FN]. Furthermore, it yielded an average duration of 28.85 seconds, a best-fit value of 0.33, and a standard deviation of 0.022. The evaluation outcomes for accuracy, precision, *f*_score, recall, MCC, and *g*_mean were 0.6772, 0.6752, 0.6829, 0.6920, 0.3541, and 0.6832, respectively. To address the first of the initial objectives, the results of the GAHA-SVM optimization are compared with those obtained from the conventional SVM grid search optimization. The comparison of optimization results can be found in Table 6, while the evaluation metrics are compared in Figure 4.  Figure 5 gives the box plot of objective functions of GAHA-SVM and the 13 competitors across the 15 independent runs, and Table 7 shows the summary statistics of their objective function over those runs.

**Figure 6:**
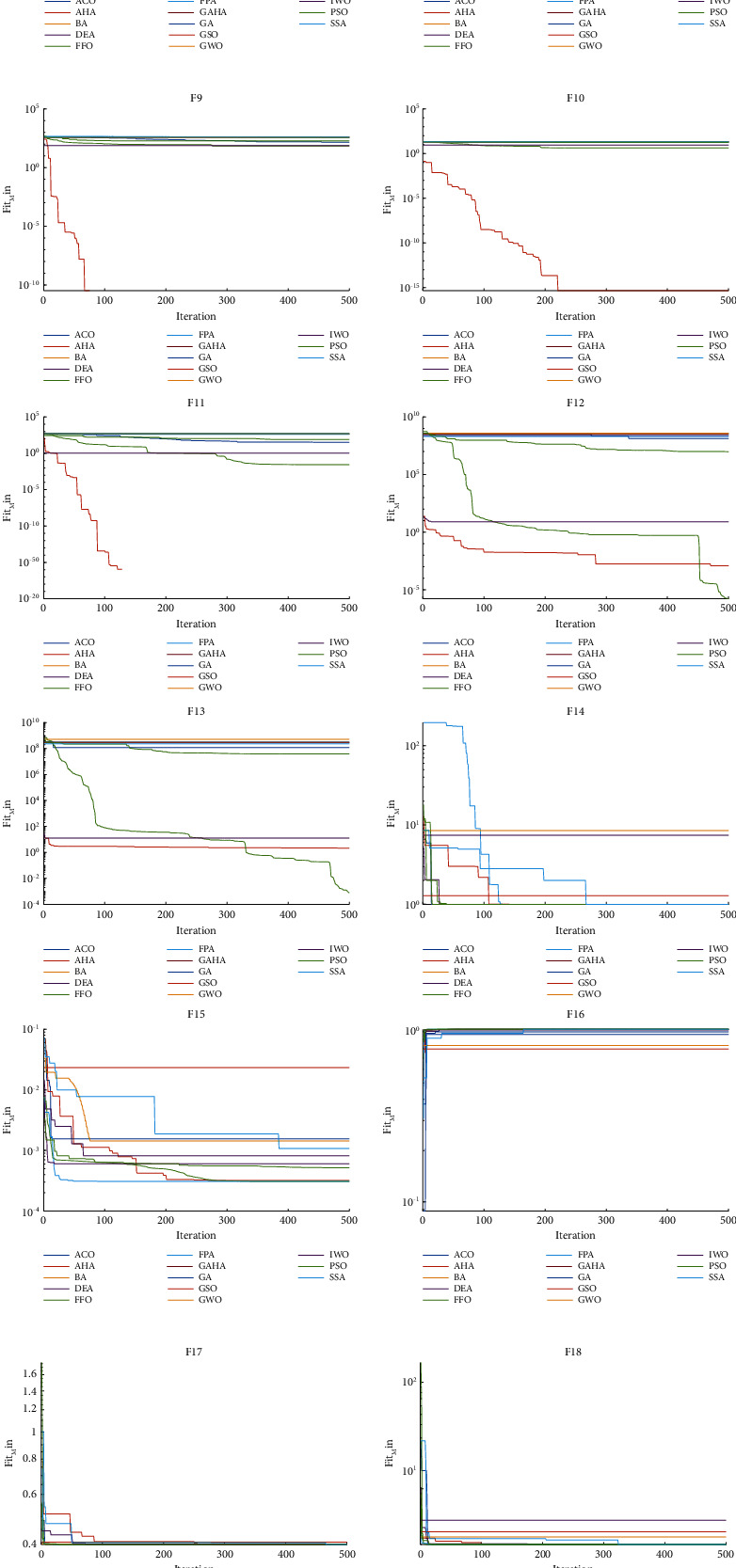
Convergence curves of GAHA-SVM and 13 competitor algorithms over the 23 benchmark functions.

The performance of the GAHA-SVM algorithm and 13 other competing algorithms was assessed using 23 standard benchmark functions outlined in Section 4.6. The mean and standard deviation obtained from these tests are presented and compared in Table 8, while the comparative runtime in seconds is provided in Table 9. Additionally, the convergence curves of these benchmark functions are illustrated in Figure 6. To further evaluate the GAHA-SVM algorithm, it was compared with the 13 competitor algorithms using the Wilcoxon rank-sum test, as described in Section 4.7. The results of these tests are presented in Table 10.

### 5.2. Discussion

A combination of metrics is necessary to comprehensively view a model's performance. The study used six metrics (accuracy, precision, recall, *f*_score, *g*score, and MCC) to assess model and competitor performance, with each model providing unique insights.

#### 5.2.1. Evaluation Metrics Discussed

The GAHA-SVM model achieved an accuracy score of 0.9986, indicating that 99.86% of the predictions made were accurate, regardless of whether the users were fraudulent or nonfraudulent. On the other hand, precision measures the proportion of true positive predictions to the total predicted positives. The model scored 0.9971 on this metric, indicating that 99.71% of the instances predicted as fraudulent were fraudulent. The recall (sensitivity) metric, which measures the proportion of true positive predictions to the actual positives, yielded a score of 1, indicating that 100% of the fraudulent instances were correctly classified.

Precision and recall are often seen as conflicting measures because improving one usually leads to a decrease in the other. Precision aims to minimize false positives, while recall focuses on reducing false negatives. To address this issue, the *f*_score was used as one of the evaluation metrics. The *f*_score provides a balanced assessment of precision and recall by calculating their harmonic mean. It considers both false positives and false negatives and is a decisive metric. It is beneficial in instances of class imbalance or when both precision and recall are equally important and cannot be compromised. The model achieved a *f*_score of 0.9986, which translates to 99.86%. This indicates a strong balance between precision and recall. Generally, a *f*_score above 0.8 is considered quite good, and the model's *f*_score of 0.9986 suggests that it performs well in correctly identifying positive cases (precision) and capturing a high proportion of actual positive cases (recall). The Matthews correlation coefficient (MCC) is a scoring system that ranges from −1 to +1. A score of +1 signifies perfect classification, while a score of 0 indicates no better than random classification. On the other hand, a score of −1 suggests complete disagreement between the predictions made and the actual observations. An MCC score of 0.9972 indicates a reasonably strong positive correlation between the predictions and the actual classifications. This suggests that the model is making predictions that align with the true outcomes. The *g*score combines precision and recall using the geometric mean, placing more emphasis on the lower of the two values. This makes it a crucial metric in cases where one precision or recall is significantly lower than the other. *G*score values closer to 1 indicate strong performance in both precision and recall. The model's *g*score of 0.9987 or 99.87% suggests that the model achieved a good balance between precision and recall. It effectively balances the trade-off between the two by making accurate positive predictions and capturing a high proportion of actual positive cases.

As illustrated in Figure 2, GAHA-SVM demonstrated superior performance across all six evaluation metrics when compared to thirteen other competitor algorithms. By leveraging the strengths of AHA and GA, a balance was struck between exploration and exploitation, resulting in the identification of the optimal hyperparameters [5.7531, 0.279] and superior performance across all metrics. The competitor algorithms, except for GA, utilize deterministic methods to update solutions which keep recycling poor solutions. GAHA offered a diversion by the stochastic generations of new solutions that brought diversity to the search space and enabled the algorithm to find a better optimum than the others. Table 5 reveals that the algorithm is outperformed by ten algorithms and outperforms three algorithms (ACO, GA, and FPA which take 15792.09, 13,384.61, and 15,929 seconds, respectively) in terms of time complexity. The bat algorithm (BA) clocks the fastest average time at 46.04 seconds on average. Using an echolocation mechanism for local and global search enables it to converge to optimal solutions speedily. Both Figure 2 and Table 5 demonstrate that the hybrid GAHA-SVM surpassed the AHA-SVM in all aspects, except for the AHA-SVM's average timing sequence of 555.82 seconds, which bested the GAHA-SVM's timing of 4,656.18 seconds. The AHA is quicker but fails to obtain the optimum solution and suffers premature convergence. The GAHA-SVM convergence is an improvement on the AHA-SVM algorithm as depicted in Figure 3. The hybrid approach enhanced overall performance contributing to improved convergence. By altering the frequency of exploration, GAHA offered a better balance between exploration and exploitation. Its ability to generate new candidate solutions to replace the worse solutions in the search space by utilizing the GA crossover and mutation operators gave the algorithm the ability to find better optimum values since diverse solutions were being added to the search space augmenting the exploration and ensuring the algorithm was not stuck in the local optimum.

The GAHA-SVM model optimization outperforms the traditional grid search of the SVM, as shown in Figure 4 and Table 6. The traditional grid search returned far worse scores across all six evaluation metrics and had a worse convergence offering only a better time complexity. The GAHA-SVM performed so well in contrast to the SVM because metaheuristic algorithms are designed to explore a broader search space more efficiently, by learning from past solutions, while the SVM grid search tries to find every possible combination of hyperparameters. This made the GAHA more efficient, leading to a faster convergence and superior solutions. In comparison with the SVM, it is less likely to get stuck in the local optima, which was crucial to finding near-optimal hyperparameters. Another key aspect that enabled the GAHA-SVM to edge the SVM is the adaptability in the GAHA-SVM's search strategy which is lacking in SVM's conventional grid search. When the GAHA-SVM algorithm threatened to get stuck in the local optima, the genetic operators would diversify the search space helping it escape and search for more promising solutions. This ability to dynamically adjust the exploration-exploitation balance during the optimization process helped the GAHA-SVM algorithm explore promising regions more intensively, refine solutions as needed, and arrive at a better optimum.

#### 5.2.2. Objective Functions over 15 Runs Discussed

The summary statistics in Table 7 show GAHA-SVM as the best performer. It achieved the lowest mean, median, standard deviation, and variance, indicating superior reliability in minimizing the objective function across all runs. By contrast, AHA-SVM performed moderately with higher mean and median values and greater variance, suggesting less stability. GA-SVM, GSO-SVM, PSO-SVM, and ACO-SVM exhibited higher variability and less consistent performance. At the same time, CS-SVM, BA-SVM, and SSA-SVM performed relatively better but still lacked the consistency of GAHA-SVM. FFO-SVM and DEA-SVM's high median and standard deviation as well as IWO-SVM and FPA-SVM's high standard deviation and variance showed that their performance was less reliable.

The box plot of the algorithms' objective functions across the independent run given in Figure 5 shows that GAHA-SVM is highly robust and has minimal variance. It consistently achieved an objective function value of zero in all but one run where it had an objective function of 0.0179. AHA-SVM showed moderate variance with values ranging from about 0.0193 to 0.4429, but with a relatively low median. GA-SVM and GSO-SVM had a broader range which indicated higher variability and less stability. The remaining algorithms (CS-SVM, PSO-SVM, GWO-SVM, FFO-SVM, BA-SVM, SSA-SVM, DEA-SVM, IWO-SVM, FPA-SVM, and ACO-SVM) had varied performances, often with higher medians and interquartile ranges, indicating less consistent performance compared to GAHA-SVM.

#### 5.2.3. Benchmark Functions Discussed

The mean of the minimal fitness and standard deviations for the algorithms over the 15 runs were recorded during the benchmark functions tests. In instances where more than one algorithm got the same minimum fitness mean, the algorithm with the lower standard deviation was considered as the winner of that test function. Across the 23 benchmark functions, the results in Table 8 and the convergence curves given in Figure 6 show that the GAHA-SVM algorithm outperformed or attained the same optimum value as some of the other algorithms in 12 of the 23 benchmark functions which are around 52 percent of the total benchmark functions. It also showed better convergence in those same set of 12 test functions compared or equal to its competitors as can be observed from the corresponding convergence curves. Its best performances came in the unimodal and multimodal test functions where it won 12 of the combined 13 but struggled consistently across the 10 fixed-dimensional multimodal functions. The GAHA-SVM algorithm attained the joint optimum value in 7 out of 7 unimodal benchmark functions along with BA and GA. This suggests that our GAHA-SVM algorithm excels in scenarios where the solution space of the problem contains a singular global optimum. Furthermore, it showcases the algorithm's reliability and effectiveness in solving simple optimization problems characterized by objective functions with single peaks. In addition, the GAHA-SVM algorithm attained the joint best optimum value in 5 of the 6 multimodal benchmark functions along with BA and GA. This indicates that the algorithm demonstrates robustness in handling complex multipeaked solution landscapes. This ability to perform optimization and converge to optimum solutions in the presence of multiple optima highlights the GAHA-SVM's versatility across a broader range of problems when compared to its competitors. While the GAHA-SVM algorithm performed exceptionally well in the unimodal and all but one multimodal benchmark functions, it faced challenges in the fixed-dimensional multimodal benchmark functions failing to come up top in a single one. The flower pollination algorithm, FPA, emerged as the best performing in 5 of those 10 functions (f15, f18, f21, f22, and f23) while the particle swarm algorithm performed best in 4 of the 10 (f14, f16, f17, and f20). The results from the fixed-dimensional multimodal benchmark function test indicate that in problems where the solution space is both multipeaked and fixed dimensional, the FPA might be more effective compared to our GAHA-SVM algorithm.

Evidenced by the results across all three sets of benchmark functions, it can be concluded that the GAHA-SVM algorithm excels in the unimodal and almost all multimodal scenarios showcasing its effectiveness across a wide array of optimization challenges. However, the challenges posed by the fixed-dimensional multimodal benchmark functions where it is outperformed by the FPA indicate that while the GAHA-SVM is powerful, its performance might be influenced by the specific characteristics of the optimization problem at hand.

In the timing sequence for the 23 benchmark functions given by Table 9, it is seen that the GAHA-SVM despite acing 12 of the 23 benchmark functions required more time than the other algorithms with the grey wolf optimizer (GWO) being the fastest algorithm across all 23 benchmark functions. It is noteworthy that the GWO's conceptual simplicity with very few parameters to set makes it computationally lightweight allowing for faster implementation and execution. On the contrary, the GAHA-SVM which is a hybrid algorithm combines a mix of optimization techniques from genetic algorithm with artificial hummingbird algorithm with each having its time complexity. Since both GA and AHA components are time-intensive, their combination resultantly led to a comparatively higher time complexity compared to the other algorithms.

#### 5.2.4. Statistical Test Discussed

The study compared the evaluation metrics of GAHA-SVM and 13 competitor algorithms to determine if their results had the same distribution over 15 runs. A significant statistical difference indicated that the observed differences were unlikely to have occurred by random chance. Out of 78 tests, 70 had a *h* value equal to 1, indicating a significant statistical difference between GAHA-SVM and the competitor algorithm. In the context of the research, a significant statistical difference was observed 90% of the time between GAHA-SVM and the competitor algorithms, while a significant difference did not occur 10% of the time. The *p* value was used in addition to access the evidence against the null hypothesis. A similar pattern was observed with results showing that 90% of the time there was a significant statistical difference and 10% of the time the null hypothesis was rejected. From Table 10, one can see that GAHA is significantly different from AHA in terms of both *h* value and *p* value across every metric except recall. This demonstrates that the observed superiority of GAHA-SVM is a reliable and consistent pattern, not due to random chance.

## 6. Limitations and Recommendations

One limitation of this study is the model's generalizability. The study utilized historical electricity consumption data from a specific community in Liberia, which might not fully represent diverse scenarios. The dataset used may have inherent biases that could affect the model's performance when applied to different regions or populations. This limitation is understandable given the bureaucratic challenges researchers face in accessing data from authorities in the country. Despite this limitation, the study extracted valuable insights from the available data and demonstrated the potential of GAHA in a real-world context. It is recommended that a broader dataset be explored for future research. Another drawback is the time complexity of the GAHA algorithm. The hybridization of GA and AHA introduced additional computational overhead, which can be time-consuming and resource-intensive, especially for large datasets. This can hinder the algorithm's scalability and practical deployment in real-time applications. Future research should focus on optimizing the algorithm to reduce its time complexity without compromising efficiency. Lastly, the study focuses solely on the classification of fraudulent and nonfraudulent electricity consumers. While the results are promising, the algorithm's performance on other types of classification problems remains unexplored. This limitation suggests a need for further research to test GAHA's applicability and effectiveness across different domains and datasets.

## 7. Conclusion

This study proposed a hybrid genetic artificial hummingbird algorithm (GAHA) with the algorithm used to optimize the support vector machine's (SVM) *C* and gamma hyperparameters. In the GAHA, the exploration stage of the AHA is enhanced using the GA's mutation and crossover operators. This variation on the AHA improves its exploration phase and gives balance to the exploration and exploitation. The population is diversified by the variations introduced by those operators enabling the algorithm to escape the local optima and avoid premature convergence. The GAHA was tested against 13 advanced optimization algorithms in the 23 optimization benchmark functions. It outperformed or equaled the performance of the other algorithms in 12 of the 23 benchmark functions. Also, the algorithm showed a 90% percent significant statistical difference in the paired Wilcoxon rank-sum test between itself and the competitors. The algorithm was used to classify fraudulent and nonfraudulent electricity consumers using labeled historical electricity consumption data. The GAHA outperformed all of the 13 competitor algorithms on the 6 evaluation metrics. The potential of the GAHA algorithm in real-world applications is confirmed by its observed superiority and accelerated convergence. This aligns with the research by Ali et al. [21] and Ibrahim et al. [22], emphasizing the computational challenges of traditional grid search and advocating for genetic algorithms in hyperparameter tuning. Abbas et al.'s research [36] further supports the inferiority of grid search compared to metaheuristic search. Moreover, the hybridization of genetic algorithm mutation operators with the artificial hummingbird algorithm represents a novel approach to tackling the imbalance between exploration and exploitation, as well as the issue of local optima convergence. The improved optimization results achieved by the GAHA algorithm substantiate the effectiveness of this approach, consistent with Zhao et al. [8], Abd Elaziz et al. [25], and Dobrojovic et al.'s [38] emphasis on the benefits of hybridization for enhanced performance. GAHA improved SVM performance for power theft detection by finding optimal values for C and gamma. Through enhancing exploration and balancing exploitation, GAHA accuracy of 99.86% surpassed the accuracy of 81% achieved by SVM [27]. GAHA genetic operators can ensure diverse exploration and evaluation metrics similar to how feature engineering and preprocessing did for those in [28, 29]. Unlike the method in [30] which relied on a hybrid neural network, GAHA combines genetic operators with artificial hummingbird algorithm to achieve optimum results. Furthermore, the binary classification done in this research returns optimal results, in contrast with the work of Salb et al. [31] which struggled noticeably in binary classification.

The algorithm can be practically deployed to grids to predict power theft. Future research could focus on reducing GAHA's time complexity, improving its performance on fixed-dimensional multimodal benchmark functions, and expanding the algorithm to other domains such as medical diagnostics, financial prediction, and image recognition, all of which require hyperparameter optimization and classification. Possible future applications include the following:

1. *Medical Diagnostics*. GAHA may be used to classify medical conditions based on patient data, through the optimization of hyperparameters in machine learning models to increase diagnostic accuracy and illness detection.
2. *Financial Prediction*. By optimizing predictive models, the algorithm may be utilized in the prediction of stock prices and credit ratings or the detection of fraudulent transactions in financial systems.
3. *Image Recognition*. GAHA can potentially be applied to computer vision tasks, optimizing the model's parameters for improved accuracy and reliability in object detection and recognition systems.

## Figures and Tables

**Algorithm 1:**
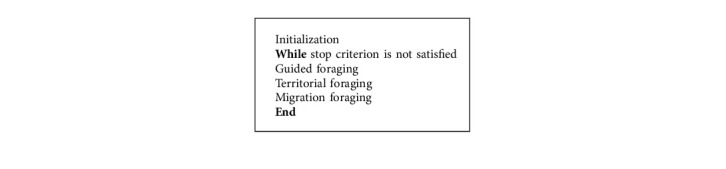
General structure of AHA.

**Algorithm 2:**
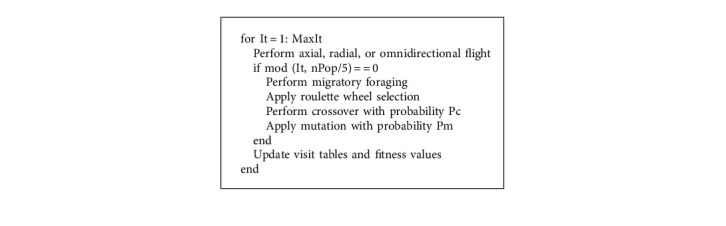
Migratory foraging with genetic operators.

**Algorithm 3:**
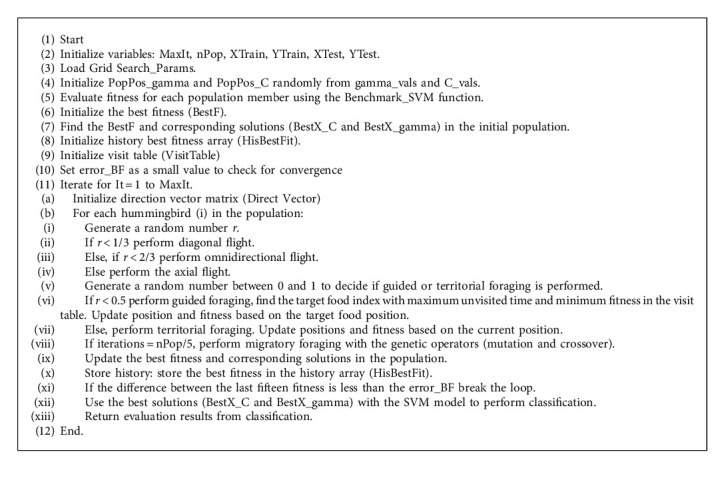
GAHA-SVM's pseudocode.

**Algorithm 4:**
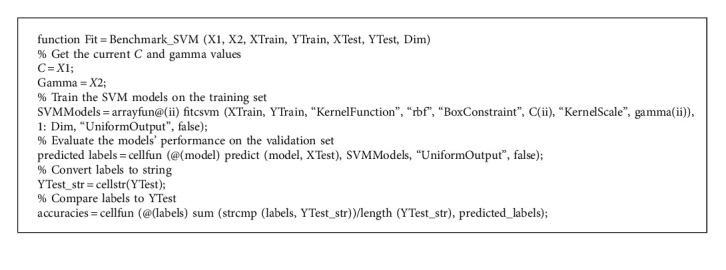
Fitness function formulation.

**Table 1:** Dataset fragment.

Inputs		Output
Billing (in USD)	Global active power (in KWh)	Status
25	71.43	Nonfraudulent
15	42.86	Nonfraudulent
50	142.86	Nonfraudulent
30	100.71	Fraudulent
25	86.43	Fraudulent

**Table 2:** Parameter setting for GAHA-SVM and the 13 competitor algorithms.

Algorithm	Parameters	Value	Hyperparameter range
DEA-SVM	Number of dolphins	20	*C* = [2^−5^, 2^3^], gamma = [2^−2^, 2^3^]
	Search area	0.5	
	Detection range	0.1	
	Iterations	100	

BA-SVM	Number of bats	20	*C* = [2^−5^, 2^3^], gamma = [2^−2^, 2^3^]
	Loudness	1	
	Pulse rate	0.5	
	Loudness scaling factor	0.9	
	Pulse scaling factor	0.9	
	Iterations	100	

FFO-SVM	Number of fruit flies	20	*C* = [2^−5^, 2^3^], gamma = [2^−2^, 2^3^]
	Iterations	100	

SSA-SVM	Number of squirrels	20	*C* = [2^−5^, 2^3^], gamma = [2^−2^, 2^3^]
	Minimum gliding distance	1.11	
	Maximum gliding distance	0.5	
	Gliding constant	1.9	
	Iterations	100	

GA-SVM	Population size	20	*C* = [2^−5^, 2^3^], gamma = [2^−2^, 2^3^]
	Crossover rate	0.8	
	Mutation rate	0.1	
	Number of elites	2	
	Iterations	100	

FPA-SVM	Number of flowers	20	*C* = [2^−5^, 2^3^], gamma = [2^−2^, 2^3^]
	Pollination probability	0.8	
	Pollen dispersal coefficient	0.5	
	Flower competition strength	1	
	Flower attraction coefficient	2	
	Iterations	100	

CS-SVM	Number of cuckoos	20	*C* = [2^−5^, 2^3^], gamma = [2^−2^, 2^3^]
	Probability of egg discovery	0.25	
	Step size	0.5	
	Iterations	100	

GAHA-SVM	Number of hummingbirds	20	*C* = [2^−5^, 2^3^], gamma = [2^−2^, 2^3^]
	Crossover rate	0.8	
	Mutation rate	0.1	
	Iterations	100	

AHA-SVM	Number of hummingbirds	20	*C* = [2^−5^, 2^3^], gamma = [2^−2^, 2^3^]
	Iterations	100	

ACO-SVM	Number of ants	20	*C* = [2^−5^, 2^3^], gamma = [2^−2^, 2^3^]
	Pheromone importance factor	1	
	Heuristic importance factor	2	
	Pheromone evaporation rate	0.2	
	Iterations	100	

GWO-SVM	Number of grey wolves	20	*C* = [2^−5^, 2^3^], gamma = [2^−2^, 2^3^]
	Iterations	100	

PSO-SVM	Number of particles	20	*C* = [2^−5^, 2^3^], gamma = [2^−2^, 2^3^]
	Cognitive parameters	2	
	Social parameters	2	
	Inertia weight	0.7	
	Iterations	100	

IWO-SVM	Number of weeds	20	*C* = [2^−5^, 2^3^], gamma = [2^−2^, 2^3^]
	Probability of reproduction	0.8	
	Probability of elimination and dispersion	0.1	
	Iterations	100	

GSO-SVM	Number of glowworm	20	*C* = [2^−5^, 2^3^], gamma = [2^−2^, 2^3^]
	Sensing range	3	
	Glow coefficient	0.4	
	Iterations	100	

**Table 3:** Data summary.

Nature of the data	
Rows	60,666
Columns	13
Majority instances	42,466
Minority instances	18,200
Percentage of majority instances	70%
Percentage of minority instances	30%

Data type	Time series

Predictor variables	2
Response variables	1
Randomly selected instances used for classification models	7276
Number of instances for training	5821
Number of instances for validation	728
Number of instances for testing	727

**Table 4:** Effects of data balancing.

Data preprocessing	Original	After
Total dataset instances	60,666	36,380
Nonfraudulent instances	42,466	18,190
Fraudulent instances	18,200	18,190
Percentage of nonfraudulent	70%	50%
Percentage of fraudulent	30%	50%
Rows with missing values	32	0
Outliers	24	0

**Table 5:** Comparison of GAHA-SVM and competitor algorithm optimization results.

Algorithm	Best hyperparameters [C, Gamma]	Confusion matrix [TP FN, FP TN]	Average time (S)	Best fit	Standard deviation
GAHA-SVM	[5.753, 0.279]	[347 0, 1 379]	4656.18	0.0016	0.055
AHA-SVM	[3.4498, 1.2976]	[288 66, 15 358]	555.82	0.11142	0.036702
DEA-SVM	[8261.76, 37.605]	[332 36, 150 209]	365.1	0.2554	5.7332*E* − 17
BA-SVM	[3.7007, 1.7833]	[345 26, 90 266]	46.04	0.000	0.0902
FFO-SVM	[8261.7, 37.605]	[319 38, 185 185]	374.98	0.307	1.15*E* − 16
SSA-SVM	[3.2288, 2.225097]	[344 39, 79 265]	358.47	0.1799	0.01422
IWO-SVM	[3.453, 3.1856]	[308 69, 62 288]	2067.61	0.1799	0.0031
GSO-SVM	[8261.76, 37.605]	[332 29, 150 209]	2918.79	0.2554	5.7332*E* − 17
PSO-SVM	[7745.42, 35.27]	[339 19, 159 210]	3663.43	0.2448	1.1466*E* − 16
GWO-SVM	[20.7214, 0.0024]	[332 23, 146 226]	56.77	0.000	0.000
ACO-SVM	[7745.42, 35.2729]	[209 163, 45 310]	15792.09	0.2853	5.7332*E* − 17
CS-SVM	[3.4763, 2.3049]	[297 55, 86 289]	3716.78	0.19	0.0056719
FPA-SVM	[7745, 35.2706]	[222 140, 39 326]	15929.13	0.2466	0.000
GA-SVM	[14992.18, 32.201]	[151 225, 15 336]	13384.61	0.000	0.000

**Table 6:** GAHA-SVM's optimization results compared to SVM's grid search.

Algorithm	Best hyperparameters [C, Gamma]	Confusion matrix [TP FN, FP TN]	Average time (S)	Best fit	Standard deviation
GAHA-SVM	[5.753, 0.279]	[347 0, 1 379]	4656.18	0.0016	0.055
SVM	[0.5, 0.4]	[253 113, 122 239]	28.85	0.3228	0.0222

**Table 7:** GAHA-SVM and competitor's objective function summary statistics across the 15 independent runs.

Algorithm	Best	Worst	Mean	Median	Standard deviation	Variance
GAHA-SVM	0	0.0179	0.001193	0	0.004622	2.14*E* − 05
AHA-SVM	0.0193	0.4429	0.1251	0.1348	0.11773	0.01386
GA-SVM	0	0.5048	0.248133	0.3494	0.233586	0.054563
GSO-SVM	0	0.4938	0.255387	0.2957	0.231879	0.053768
CS-SVM	0	0.4085	0.19468	0.2215	0.134975	0.018218
PSO-SVM	0	0.5076	0.244767	0.2847	0.226023	0.051087
GWO-SVM	0	0.5117	0.231453	0.1444	0.239284	0.0572557
FFO-SVM	0	0.5089	0.306993	0.4525	0.230589	0.053171
BA-SVM	0.0193	0.3824	0.15938	0.1706	0.102454	0.010497
SSA-SVM	0	0.3301	0.163253	0.1376	0.127947	0.01637
DEA-SVM	0	0.4938	0.255387	0.2957	0.231879	0.053768
IWO-SVM	0	0.4718	0.17992	0.0757	0.175811	0.030909
FPA-SVM	0	0.4979	0.246411	0.1541	0.223722	0.050051
ACO-SVM	0	0.5117	0.285947	0.3439	0.218416	0.047705

**Table 8:** Comparison of optimization results for GAHA-SVM and competitors across the 23 benchmark functions.

Function	GAHA-SVM	GSO-SVM	CS-SVM	PSO-SVM	AHA-SVM	GWO-SVM	GA-SVM	FFO-SVM	BA-SVM	SSA-SVM	DEA-SVM	IWO-SVM	FPA-SVM	ACO-SVM
*F*1 Mean	0.00*E + *00	6.31*E + *04	6.49*E + *04	2.35*E + *04	−2.28*E − *24	6.16*E + *04	0.00*E + *00	1.03*E − *02	0.00*E + *00	6.75*E + *04	6.46*E + *04	2.91*E + *02	6.82*E + *04	5.34*E + *03
Std	0.00*E + *00	5.97*E + *03	5.30*E + *03	1.04*E + *04	8.84*E − *24	8.16*E + *03	0.00*E + *00	1.91*E − *02	0.00*E + *00	5.60*E + *03	5.92*E + *03	1.54*E + *01	4.83*E + *03	2.81*E + *03

*F*2 Mean	0.00*E + *00	6.08*E + *12	9.10*E + *01	1.72*E + *02	−2.77*E − *34	NaN	0.00*E + *00	1.25*E + *00	0.00*E + *00	1.05*E + *10	4.87*E + *08	2.71*E + *01	1.97*E + *12	5.40*E + *06
Std	0.00*E + *00	1.24*E + *13	1.08*E + *01	2.02*E + *02	8.36*E − *34	NaN	0.00*E + *00	2.42*E + *00	0.00*E + *00	1.94*E + *10	7.73*E + *08	5.28*E + *00	4.02*E + *12	1.50*E + *07

*F*3 Mean	0.00*E + *00	1.40*E + *05	1.02*E + *05	6.03*E + *04	−1.44*E − *29	1.21*E + *05	0.00*E + *00	3.29*E + *03	0.00*E + *00	1.02*E + *05	8.46*E + *04	4.11*E + *04	1.27*E + *05	4.76*E + *04
Std	0.00*E + *00	3.06*E + *04	2.97*E + *04	2.00*E + *04	5.70*E − *29	3.36*E + *04	0.00*E + *00	2.22*E + *03	0.00*E + *00	2.24*E + *04	1.55*E + *04	1.14*E + *04	2.77*E + *04	7.21*E + *03

*F*4 Mean	0.00*E + *00	8.54*E + *01	8.58*E + *01	8.38*E + *01	2.54*E − *30	8.47*E + *01	0.00*E + *00	2.15*E + *01	0.00*E + *00	8.71*E + *01	8.64*E + *01	4.43*E + *00	8.42*E + *01	7.24*E + *01
Std	0.00*E + *00	4.56*E + *00	3.08*E + *00	5.54*E + *00	7.25*E − *30	3.46*E + *00	0.00*E + *00	7.73*E + *00	0.00*E + *00	3.10*E + *00	3.74*E + *00	1.68*E − *01	4.87*E + *00	7.23*E + *00

*F*5 Mean	0.00*E + *00	2.51*E + *08	1.97*E + *08	7.09*E + *07	2.33*E − *01	NaN	0.00*E + *00	3.65*E + *03	0.00*E + *00	2.32*E + *08	2.22*E + *08	1.91*E + *05	2.29*E + *08	1.09*E + *08
Std	0.00*E + *00	3.11*E + *07	1.90*E + *07	2.99*E + *07	4.90*E − *01	NaN	0.00*E + *00	7.37*E + *03	0.00*E + *00	4.18*E + *07	5.34*E + *07	3.46*E + *04	5.74*E + *07	2.04*E + *07

*F*6 Mean	0.00*E + *00	6.45*E + *04	6.90*E + *04	2.33*E + *04	−4.60*E − *02	6.47*E + *04	0.00*E + *00	1.81*E + *03	0.00*E + *00	6.54*E + *04	6.59*E + *04	2.91*E + *02	6.67*E + *04	4.37*E + *03
Std	0.00*E + *00	3.44*E + *03	5.13*E + *03	1.19*E + *04	1.89*E − *01	4.82*E + *03	0.00*E + *00	1.07*E + *03	0.00*E + *00	5.93*E + *03	4.40*E + *03	1.34*E + *01	5.76*E + *03	1.75*E + *03

*F*7 Mean	0.00*E + *00	1.12*E + *02	2.55*E + *01	5.23*E + *01	−6.34*E − *03	NaN	0.00*E + *00	5.05*E − *01	0.00*E + *00	1.22*E + *02	1.11*E + *02	1.85*E + *00	1.04*E − *01	1.25*E + *00
Std	0.00*E + *00	2.33*E + *01	1.25*E + *01	2.69*E + *01	5.59*E − *02	NaN	0.00*E + *00	3.04*E − *01	0.00*E + *00	1.76*E + *01	1.79*E + *01	6.34*E − *01	4.68*E − *02	7.45*E − *01

*F*8 Mean	0.00*E + *00	−2.19*E + *03	−2.46*E + *03	−2.85*E + *88	−7.29*E + *09	−1.11*E + *126	−1.00*E + *06	−1.59*E + *150	−3.14*E + *03	−6.88*E + *03	−8.88*E + *03	−2.69*E + *03	−2.24*E + *03	−3.28*E + *03
Std	0.00*E + *00	3.68*E + *02	3.68*E + *02	5.23*E + *88	2.32*E + *10	4.32*E + *126	7.95*E + *05	4.20*E + *150	3.25*E + *02	5.01*E + *02	6.92*E + *02	5.99*E + *02	3.94*E + *02	5.07*E + *02

*F*9 Mean	0.00*E + *00	4.27*E + *02	2.48*E + *02	2.92*E + *02	2.60*E − *10	4.28*E + *02	0.00*E + *00	1.12*E + *02	0.00*E + *00	4.37*E + *02	4.29*E + *02	1.16*E + *02	3.76*E + *02	1.78*E + *02
Std	0.00*E + *00	1.64*E + *01	2.31*E + *01	4.04*E + *01	1.12*E − *09	2.36*E + *01	0.00*E + *00	2.65*E + *01	0.00*E + *00	2.04*E + *01	2.16*E + *01	1.91*E + *01	3.32*E + *01	4.21*E + *01

*F*10 Mean	0.00*E + *00	2.05*E + *01	1.95*E + *01	1.88*E + *01	−5.05*E − *18	2.05*E + *01	0.00*E + *00	1.45*E + *01	0.00*E + *00	2.06*E + *01	2.05*E + *01	9.92*E + *00	2.06*E + *01	2.00*E + *01
Std	0.00*E + *00	2.60*E − *01	2.05*E − *01	1.29*E + *00	1.28*E − *16	3.66*E − *01	0.00*E + *00	3.58*E + *00	0.00*E + *00	1.35*E − *01	1.94*E − *01	5.79*E − *01	1.82*E − *01	2.24*E − *01

*F*11 Mean	0.00*E + *00	5.83*E + *02	5.93*E + *02	2.45*E + *02	8.80*E − *10	5.91*E + *02	0.00*E + *00	8.92*E − *01	0.00*E + *00	5.84*E + *02	5.70*E + *02	1.07*E + *00	5.96*E + *02	6.10*E + *01
Std	0.00*E + *00	7.00*E + *01	7.53*E + *01	1.26*E + *02	3.07*E − *09	4.80*E + *01	0.00*E + *00	1.58*E + *00	0.00*E + *00	5.30*E + *01	5.83*E + *01	7.34*E − *03	3.96*E + *01	1.73*E + *01

*F*12 Mean	0.00*E + *00	5.11*E + *08	4.16*E + *08	2.46*E + *08	−7.17*E − *01	NaN	0.00*E + *00	4.75*E − *01	0.00*E + *00	5.05*E + *08	5.66*E + *08	1.18*E + *01	5.44*E + *08	1.99*E + *08
Std	0.00*E + *00	7.27*E + *07	1.08*E + *08	1.55*E + *08	9.51*E − *01	NaN	0.00*E + *00	5.00*E − *01	0.00*E + *00	9.03*E + *07	6.43*E + *07	1.42*E + *00	1.11*E + *08	2.75*E + *07

*F*13 Mean	0.00*E + *00	5.52*E + *08	4.48*E + *08	2.49*E + *08	5.25*E − *01	NaN	0.00*E + *00	1.33*E + *01	0.00*E + *00	4.68*E + *08	4.23*E + *08	2.12*E + *01	5.12*E + *08	1.83*E + *08
Std	0.00*E + *00	1.11*E + *08	7.83*E + *07	2.25*E + *08	7.86*E − *01	NaN	0.00*E + *00	2.64*E + *01	0.00*E + *00	1.06*E + *08	1.50*E + *08	2.06*E + *00	1.06*E + *08	4.13*E + *07

*F*14 Mean	0.00*E + *00	6.44*E + *01	1.64*E + *00	9.98*E − *01	−2.00*E + *01	4.62*E + *01	0.00*E + *00	1.78*E + *00	0.00*E + *00	1.33*E + *00	9.98*E − *01	1.23*E + *01	1.52*E + *01	6.86*E + *00
Std	0.00*E + *00	7.65*E + *01	8.09*E − *01	8.39*E − *17	1.97*E + *01	4.63*E + *01	0.00*E + *00	2.51*E + *00	0.00*E + *00	3.86*E − *01	3.05*E − *04	9.32*E − *01	1.43*E + *01	5.64*E + *00

*F*15 Mean	0.00*E + *00	1.17*E − *01	1.48*E − *03	8.48*E − *04	1.29*E − *01	8.99*E − *02	0.00*E + *00	7.64*E − *04	0.00*E + *00	2.80*E − *03	1.09*E − *03	1.39*E − *03	3.15*E − *04	1.79*E − *02
Std	0.00*E + *00	5.66*E − *02	4.26*E − *04	1.93*E − *04	2.10*E − *02	1.01*E − *01	0.00*E + *00	3.61*E − *04	0.00*E + *00	1.36*E − *03	1.82*E − *04	8.93*E − *04	3.02*E − *05	1.26*E − *02

*F*16 Mean	0.00*E + *00	1.48*E − *01	−1.02*E + *00	−1.03*E + *00	1.42*E − *01	NaN	−4.12*E − *01	−1.03*E + *00	−1.02*E + *00	−1.02*E + *00	−1.03*E + *00	−9.58*E − *01	−1.03*E + *00	−5.71*E − *01
Std	0.00*E + *00	9.29*E − *01	3.18*E − *02	8.39*E − *17	7.23*E − *01	NaN	3.14*E − *01	2.06*E − *16	6.73*E − *03	9.26*E − *03	4.36*E − *04	2.46*E − *02	1.68*E − *16	3.07*E − *01

*F*17 Mean	0.00*E + *00	1.29*E + *00	3.98*E − *01	3.98*E − *01	3.65*E + *00	1.33*E + *00	0.00*E + *00	3.98*E − *01	0.00*E + *00	4.01*E − *01	3.98*E − *01	3.98*E − *01	3.98*E − *01	5.20*E − *01
Std	0.00*E + *00	6.71*E − *01	8.78*E − *05	0.00*E + *00	3.50*E + *00	1.04*E + *00	0.00*E + *00	0.00*E + *00	0.00*E + *00	4.12*E − *03	1.39*E − *04	3.81*E − *04	0.00*E + *00	1.20*E − *01

*F*18 Mean	0.00*E + *00	3.27*E + *01	3.21*E + *00	3.00*E + *00	−1.00*E + *00	NaN	0.00*E + *00	3.00*E + *00	0.00*E + *00	3.15*E + *00	3.01*E + *00	4.02*E + *01	3.00*E + *00	6.33*E + *00
Std	0.00*E + *00	3.22*E + *01	3.64*E − *01	1.76*E − *15	6.63*E − *04	NaN	0.00*E + *00	7.06*E − *15	0.00*E + *00	1.39*E − *01	5.89*E − *03	3.19*E + *01	1.09*E − *15	4.61*E + *00

*F*19 Mean	0.00*E + *00	−3.49*E + *00	−3.84*E + *00	−3.86*E + *00	8.53*E − *01	−3.49*E + *00	−3.78*E + *00	−3.86*E + *00	−6.80*E − *02	−3.85*E + *00	−3.86*E + *00	−3.65*E + *00	−3.86*E + *00	−3.83*E + *00
Std	0.00*E + *00	1.62*E − *01	2.52*E − *02	1.61*E − *15	1.26*E − *03	2.88*E − *01	9.34*E − *02	1.64*E − *15	2.87*E − *17	5.58*E − *03	7.77*E − *04	1.88*E − *01	1.41*E − *15	4.17*E − *02

*F*20 Mean	0.00*E + *00	−1.82*E + *00	−2.81*E + *00	−3.27*E + *00	6.61*E − *01	−1.80*E + *00	−2.89*E + *00	−3.25*E + *00	−5.11*E − *03	−2.89*E + *00	−3.13*E + *00	−2.32*E + *00	−3.19*E + *00	−3.00*E + *00
Std	0.00*E + *00	3.73*E − *01	1.71*E − *01	6.14*E − *02	8.56*E − *03	4.76*E − *01	1.90*E − *01	6.03*E − *02	1.80*E − *18	1.26*E − *01	5.24*E − *02	2.64*E − *01	4.89*E − *02	1.04*E − *01

*F*21 Mean	0.00*E + *00	−7.36*E − *01	−8.06*E + *00	−6.64*E + *00	3.25*E + *00	−5.07*E − *01	−1.41*E + *00	−6.48*E + *00	−2.73*E − *01	−2.69*E + *00	−5.16*E + *00	−8.16*E + *00	−8.70*E + *00	−2.89*E + *00
Std	0.00*E + *00	4.16*E − *01	2.42*E + *00	3.52*E + *00	1.70*E + *00	1.22*E − *01	1.05*E + *00	3.64*E + *00	5.75*E − *17	1.44*E + *00	1.28*E + *00	9.79*E − *01	2.37*E + *00	1.18*E + *00

*F*22 Mean	0.00*E + *00	−1.05*E + *00	−7.41*E + *00	−7.98*E + *00	2.91*E + *00	−7.48*E − *01	−1.97*E + *00	−6.77*E + *00	−2.94*E − *01	−2.47*E + *00	−4.90*E + *00	−8.61*E + *00	−8.96*E + *00	−2.98*E + *00
Std	0.00*E + *00	6.23*E − *01	2.28*E + *00	3.16*E + *00	1.59*E + *00	2.01*E − *01	1.59*E + *00	3.61*E + *00	0.00*E + *00	5.85*E − *01	1.18*E + *00	1.01*E + *00	2.61*E + *00	1.31*E + *00

*F*23 Mean	0.00*E + *00	−9.63*E − *01	−7.14*E + *00	−6.64*E + *00	2.92*E + *00	−8.99*E − *01	−1.69*E + *00	−4.31*E + *00	−3.22*E − *01	−2.77*E + *00	−5.78*E + *00	−8.41*E + *00	−9.65*E + *00	−3.12*E + *00
Std	0.00*E + *00	3.21*E − *01	2.34*E + *00	3.87*E + *00	1.95*E + *00	2.83*E − *01	6.33*E − *01	3.24*E + *00	0.00*E + *00	8.18*E − *01	1.64*E + *00	7.46*E − *01	2.01*E + *00	7.05*E − *01

**Table 9:** Comparison of GAHA-SVM and the 13 competitor algorithms' timing in seconds on the 23 benchmark functions.

Functions	GAHA-SVM	GSO-SVM	CS-SVM	PSO-SVM	AHA-SVM	GWO-SVM	GA-SVM	FFO-SVM	BA-SVM	SSA-SVM	DEA-SVM	IWO-SVM	FPA-SVM	ACO-SVM
*F*1	4.1139	0.1475	0.0147	0.0217	0.0831	0.0098	0.0900	0.0167	0.0168	0.0370	0.8184	0.0155	0.0280	4.2341
*F*2	4.3107	0.1452	0.0136	0.0232	0.0751	0.0054	0.0856	0.0185	0.0177	0.0419	0.8474	0.0156	0.0344	4.2993
*F*3	12.6158	0.2943	0.1928	0.1709	0.2444	0.0092	0.2281	0.1588	0.1744	0.1809	4.4349	0.1398	0.2804	4.5803
*F*4	4.6020	0.1449	0.0099	0.0174	0.0630	0.0044	0.0832	0.0139	0.0157	0.0332	0.8444	0.0124	0.0235	5.5609
*F*5	5.4721	0.1628	0.0330	0.0346	0.0860	0.0048	0.1021	0.0308	0.0339	0.0523	1.2926	0.0261	0.0568	5.8599
*F*6	4.6035	0.1484	0.0128	0.0244	0.0590	0.0045	0.0841	0.0160	0.0180	0.0397	1.0167	0.0133	0.0304	5.3389
*F*7	9.2995	0.2397	0.1292	0.1085	0.1508	0.0065	0.1666	0.1046	0.1090	0.1278	3.0336	0.0879	0.1812	5.2114
*F*8	5.4954	0.1626	0.0371	0.1076	0.0818	0.0059	0.1040	0.0687	0.0365	0.0568	1.6767	0.0290	0.0649	5.5428
*F*9	5.6563	0.1552	0.0303	0.0354	0.0688	0.0057	0.0950	0.0253	0.0189	0.0518	4.4363	0.0233	0.0534	5.1710
*F*10	5.4153	0.1894	0.0441	0.0382	0.0705	0.0050	0.0971	0.0279	0.0213	0.0547	4.8430	0.0259	0.0551	5.0451
*F*11	6.0644	0.2065	0.0524	0.0518	0.0841	0.0061	0.1118	0.0367	0.0364	0.0686	5.9534	0.0336	0.0777	5.0722
*F*12	17.7477	0.4001	0.3815	0.2736	0.3185	0.0120	0.3254	0.2613	0.3037	0.2931	24.5371	0.2335	0.4721	5.4944
*F*13	17.7442	0.4375	0.5284	0.2697	0.3139	0.0116	0.3204	0.2674	0.2724	0.2899	25.1354	0.2318	0.4703	5.0752
*F*14	28.9688	0.8383	0.8393	0.4655	0.5252	0.0124	0.5380	0.4645	0.4943	0.4893	28.0862	0.4052	0.8526	0.6705
*F*15	4.7919	0.1741	0.0160	0.0123	0.0559	0.0023	0.0863	0.0123	0.0141	0.0303	0.7294	0.0138	0.0315	0.7909
*F*16	5.0147	0.1723	0.0199	0.0164	0.0546	0.0021	0.0880	0.0148	0.0167	0.0340	0.7177	0.0149	0.0324	0.3968
*F*17	4.7942	0.1837	0.0118	0.0103	0.0536	0.0027	0.0961	0.0094	0.0103	0.0280	0.6067	0.0120	0.0219	0.4092
*F*18	4.9739	0.1667	0.0090	0.0067	0.0546	0.0027	0.0822	0.0074	0.0081	0.0239	0.5471	0.0081	0.0192	0.3952
*F*19	5.2183	0.2092	0.0274	0.0180	0.0633	0.0026	0.0941	0.0207	0.0206	0.0361	0.8245	0.0202	0.0419	0.6106
*F*20	5.3277	0.2228	0.0254	0.0198	0.0645	0.0032	0.0933	0.0195	0.0209	0.0378	0.9189	0.0213	0.0429	1.1659
*F*21	5.5182	0.1999	0.0281	0.0237	0.0651	0.0026	0.0969	0.0214	0.0225	0.0402	0.9421	0.0210	0.0465	0.7779
*F*22	5.7327	0.2147	0.0350	0.0276	0.0741	0.0027	0.1065	0.0279	0.0290	0.0497	1.0296	0.0265	0.0548	0.7979
*F*23	6.1233	0.2327	0.0441	0.0357	0.0759	0.0030	0.1133	0.0344	0.0381	0.0565	1.2434	0.0333	0.0723	0.8200

**Table 10:** Wilcoxon rank-sum test scores for GAHA-SVM vs. competitor algorithms on the evaluation metrics.

		GAHA-SVM vs. GSO-SVM	GAHA-SVM vs. CS-SVM	GAHA-SVM vs. PSO-SVM	GAHA-SVM vs. AHA-SVM	GAHA-SVM vs. GWO-SVM	GAHA-SVM vs. GA-SVM	GAHA-SVM vs. FFO-SVM	GAHA-SVM vs. BA-SVM	GAHA-SVM vs. SSA-SVM	GAHA-SVM vs. DEA-SVM	GAHA-SVM vs. IWO-SVM	GAHA-SVM vs. FPA-SVM	GAHA-SVM vs. ACO-SVM
Accuracy	*p* value	0.0002	0	0.002	0.002	0.0039	0.001	0.002	0.0001	0.002	0.001	0.0039	0.001	0.0002
	*h* value	1	1	1	1	1	1	1	1	1	1	1	1	1
	*z* value	1.1927	2.9534	1.3631	1.3631	0.9087	1.8743	0.6816	3.4078	1.3631	1.1927	0.9087	2.3286	1.4767

Precision	*p* value	0.0002	0	0.002	0.002	0.0156	0.0078	0.002	0.0001	0.002	0.001	0.0156	0.0156	0.001
	*h* value	1	1	1	1	1	1	1	1	1	1	1	1	1
	*z* value	1.1927	2.9534	1.3631	1.3631	-0.3976	0.1704	0.6816	3.4078	1.3631	1.1927	-0.3976	-0.3976	1.4767

Recall	*p* value	0.0797	0.0039	0.125	0.125	0.125	0.001	0.0625	0.0078	0.125	0.25	0.125	0.001	0.0005
	*h* value	0	1	0	0	0	1	0	1	0	0	0	1	1
	*z* value	-2.499	0.9087	-1.5903	-1.5903	-1.5335	1.8743	-1.5903	-0.5112	-1.5903	-2.499	-1.5335	2.3286	1.7607

*F*_score	*p* value	0.0002	0.0001	0.002	0.002	0.0039	0.001	0.002	0.0001	0.002	0.001	0.0039	0.001	0.0002
	*h* value	1	1	1	1	1	1	1	1	1	1	1	1	1
	*z* value	1.1927	2.9534	1.3631	1.3631	0.9087	1.8743	0.6816	3.4078	1.3631	1.1927	0.9087	2.3286	1.4767

*G*_mean	*p* value	0.0002	0.0001	0.002	0.002	0.0039	0.001	0.002	0.0001	0.002	0.001	0.0039	0.001	0.0002
	*h* value	1	1	1	1	1	1	1	1	1	1	1	1	1
	*z* value	1.1927	2.9534	1.3631	1.3631	0.9087	1.8743	0.6816	3.4078	1.3631	1.1927	0.9087	2.3286	1.4767

MCC	*p* value	0.0002	0.0001	0.002	0.002	0.0039	0.001	0.002	0.0001	0.002	0.001	0.0039	0.001	0.0002
	*h* value	1	1	1	1	1	1	1	1	1	1	1	1	1
	*z* value	1.1927	2.9534	1.3631	1.3631	0.9087	1.8743	0.6816	3.4078	1.3631	1.1927	0.9087	2.3286	1.4767

## Data Availability

The historical electricity consumption data used for this research are proprietary to the Liberia Electricity Corporation (LEC) and therefore restricted.
